# Molecular heterogeneous catalysts derived from bipyridine-based organosilica nanotubes for C–H bond activation†
†Electronic supplementary information (ESI) available: Experimental details, material characterization data, catalytic measurement details. See DOI: 10.1039/c7sc00713b



**DOI:** 10.1039/c7sc00713b

**Published:** 2017-04-19

**Authors:** Shengbo Zhang, Hua Wang, Mei Li, Jinyu Han, Xiao Liu, Jinlong Gong

**Affiliations:** a Key Laboratory for Green Chemical Technology of Ministry of Education , School of Chemical Engineering and Technology , Tianjin University , Collaborative Innovation Center of Chemical Science and Engineering , Tianjin 300072 , China . Email: liuxiao71@tju.edu.cn ; Email: jlgong@tju.edu.cn

## Abstract

Heterogeneous metal complex catalysts for direct C–H activation with high activity and durability have always been desired for transforming raw materials into feedstock chemicals. This study described the design and synthesis of one-dimensional organosilica nanotubes containing 2,2′-bipyridine (bpy) ligands in the framework (BPy-NT) and their post-synthetic metalation to provide highly active and robust molecular heterogeneous catalysts. By adjusting the ratios of organosilane precursors, very short BPy-NT with ∼50 nm length could be controllably obtained. The post-synthetic metalation of bipyridine-functionalized nanotubes with [IrCp*Cl(μ-Cl)]_2_ (Cp* = η^5^-pentamethylcyclopentadienyl) and [Ir(cod)(OMe)]_2_ (cod = 1,5-cyclooctadiene) afforded solid catalysts, IrCp*-BPy-NT and Ir(cod)-BPy-NT, which were utilized for C–H oxidation of heterocycles and cycloalkanes as well as C–H borylation of arenes. The cut-short nanotube catalysts displayed enhanced activities and durability as compared to the analogous homogeneous catalysts and other conventional heterogeneous catalysts, benefiting from the isolated active sites as well as the fast transport of substrates and products. After the reactions, a detailed characterization of Ir-immobilized BPy-NT *via* TEM, SEM, nitrogen adsorption, UV/vis, XPS, and ^13^C CP MAS NMR indicated the molecular nature of the active species as well as stable structures of nanotube scaffolds. This study demonstrates the potential of BPy-NT with a short length as an integration platform for the construction of efficient heterogeneous catalytic systems for organic transformations.

## Abstract 2

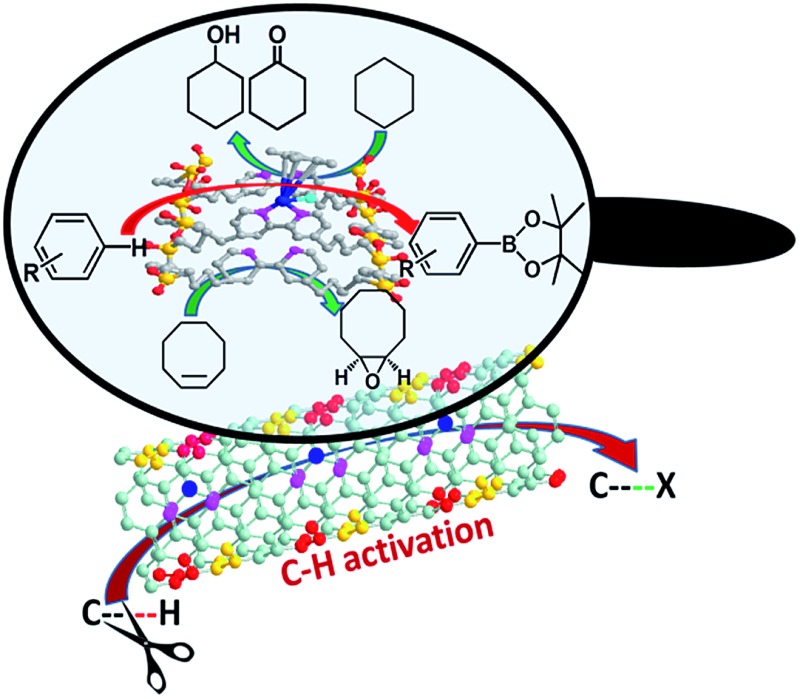
The present study described bipyridine-based organosilica nanotubes with ∼50 nm length; these nanotubes provide highly active and robust molecular iridium heterogeneous catalysts for C–H oxidation of heterocycles and cycloalkanes as well as C–H borylation of arenes.

## Introduction

The C–H activation reaction is a key reaction for the functionalization of organic compounds, which can convert raw materials or low-valued materials into feedstock and practical chemicals.^1^ Among various strategies developed to achieve this challenging goal, C–H activation catalyzed by homogeneous metal complexes has become a hot research topic in modern synthetic chemistry. Enormous progress has been made on the design and synthesis of efficient homogeneous catalysts such as Fe,^2^ Ir,^3^ Rh,^4^ Pd,^5^ and Ag.^6^

Considering the future practical applications and cost reductions,^7^ immobilization of homogeneous metal complexes on solid supports is important for catalyst recovery and reuse.^8–17^ Moreover, heterogenization has the ability to improve the stability of the catalysts as it suppresses deactivation caused by intermolecular pathways. Molecular heterogeneous catalysts also provide the facility to understand the nature of the active species, which can help to carry out mechanistic studies and optimize reaction activities through the fine-tuning of electronic or spatial effects in the molecules.

Recently, molecular heterogeneous catalysts have attracted significant research interest for C–H activation using various solids as supports, such as polymer,^18^ metal–organic frameworks (MOFs),^19^ and silica-based materials.^11,20–22^ For example, Lin *et al.* synthesized UiO-type MOFs with 2,2′-bipyridine (bpy) as an orthogonal functional fragment to form solid catalysts for both borylation of C–H bonds and *ortho*-silylation of benzylic silyl ethers.^19^ Moreover, Inagaki *et al.* and Copéret *et al.* reported an original solid, periodic mesoporous organosilicas (PMOs) with bipyridine ligands in the framework, as a unique platform for heterogeneous C–H borylation, which showed superior activity towards the homogeneous C–H borylation.^20–22^ However, with continuous reuse of the catalysts, the reaction activity gradually decreased because of the collapse of the material structure, which was also observed in the water oxidation reaction by these PMOs.^23^ Therefore, the development of novel stable scaffolds is still required for practical applications.

Organosilica nanotubes have been prepared from bridged organosilane precursors using a simple micelle-templating approach.^24–27^ These nanotubes with mesoporous diameters have distinct advantages including incorporation of various organic functionalities into the nanotube frameworks, high surface areas, easy access to active sites in the tubes, and confinement effects inside the cavity. We have recently synthesized organosilica nanotubes embedded with 2,2′-bipyridine chelating ligands^24*b*^ due to their importance in coordination and supramolecular chemistry.^28–30^ The length of the nanotubes was about several micrometers and we envisaged cropping of the long nanotubes to eliminate diffusion limitation as much as possible in heterogeneous catalysis.

**Scheme 1:**
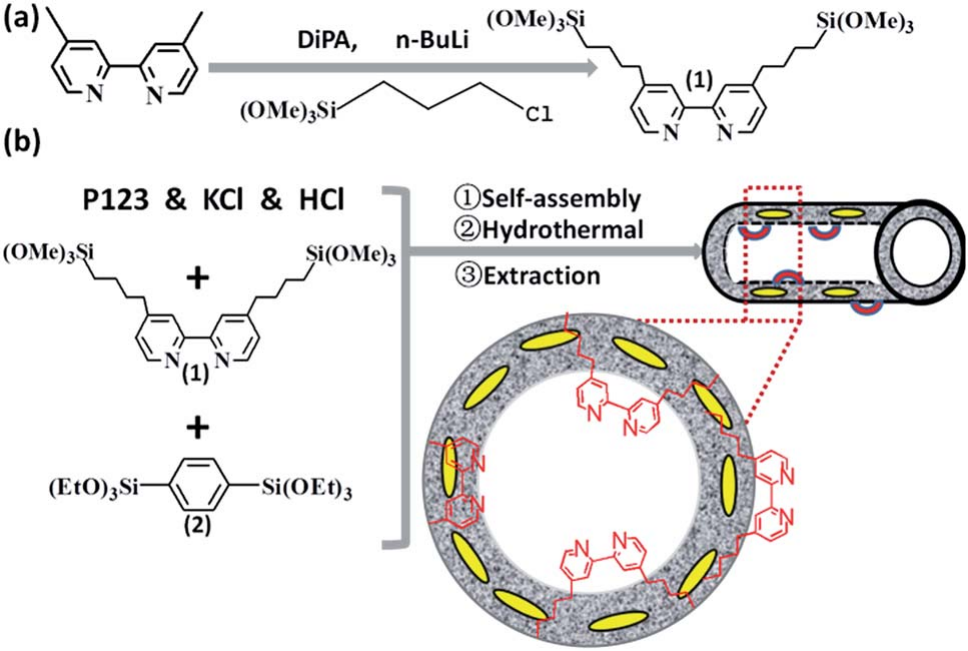
Synthetic routes for the bipyridine-based organosilica nanotubes (BPy-NT).

This study described the design and synthesis of short organosilica nanotubes with 2,2′-bipyridine ligands in the frameworks (BPy-NT); these nanotubes were prepared from organosilanes and their synthesis was easier than that reported in our previous study^24*b*^ (Scheme 1); moreover, they exhibited improved structural stability and facilitated the diffusion of reactants or products in the channels. The length of BPy-NT could be facilely controlled by adjusting the proportion of bipyridine- to benzene-bridged precursors. The shortest BPy-NT was only ∼40 nm in length with a pore diameter of ∼6 nm. Using these unique bipyridine-incorporated nanotubes as a support, we synthesized two types of molecular heterogeneous solid catalysts, IrCp*-BPy-NT (Cp* = η^5^-pentamethylcyclopentadienyl) and Ir(cod)-BPy-NT (cod = 1,5-cyclooctadiene), through post-synthetic metalation of BPy-NT with iridium precursors; these solid catalysts were characterized *via* physicochemical analysis. The C–H oxidation of heterocycles and cycloalkanes as well as directed C–H borylation of arenes reveal that the Ir-immobilized molecular heterogeneous nanotube catalysts have very high initial catalytic activities, comparable to those of the analogous homogeneous catalysts. Furthermore, the nanotube-constructed Ir catalysts exhibited significantly improved durability and recyclability, owing to the suppression of Ir-complex decomposition and aggregation pathways.

## Results and discussion

### Synthesis and characterization of BPy-NT

The bipyridine precursor (1) was prepared *via* a one-step process from commercially available reagents (Scheme 1a). 4,4′-Dimethyl-2,2′-bipyridine was first converted *via* regioselective lithiation of diisopropylamine to CH_2_^–^ anionic group and subsequently reacted with (3-chloropropyl)trimethoxylsilane to obtain the bipyridine precursor (1). The organosilica nanotubes containing bipyridine ligands were synthesized by the hydrolysis and co-condensation of bipyridine-bridged precursor (1) and 1,4-bis(triethoxysilyl)benzene (2) under acid conditions in the presence of P123 [(EO)_20_(PO)_70_(EO)_20_] as the template agent. The template-extracted nanotubes were denoted as BPy_*x*_-NT [*x* = 0.1, 0.2, and 0.3, corresponding to the molar ratio of the precursor (1) to (2) in the initial synthesis] (Scheme 1b).

**Fig. 1:**
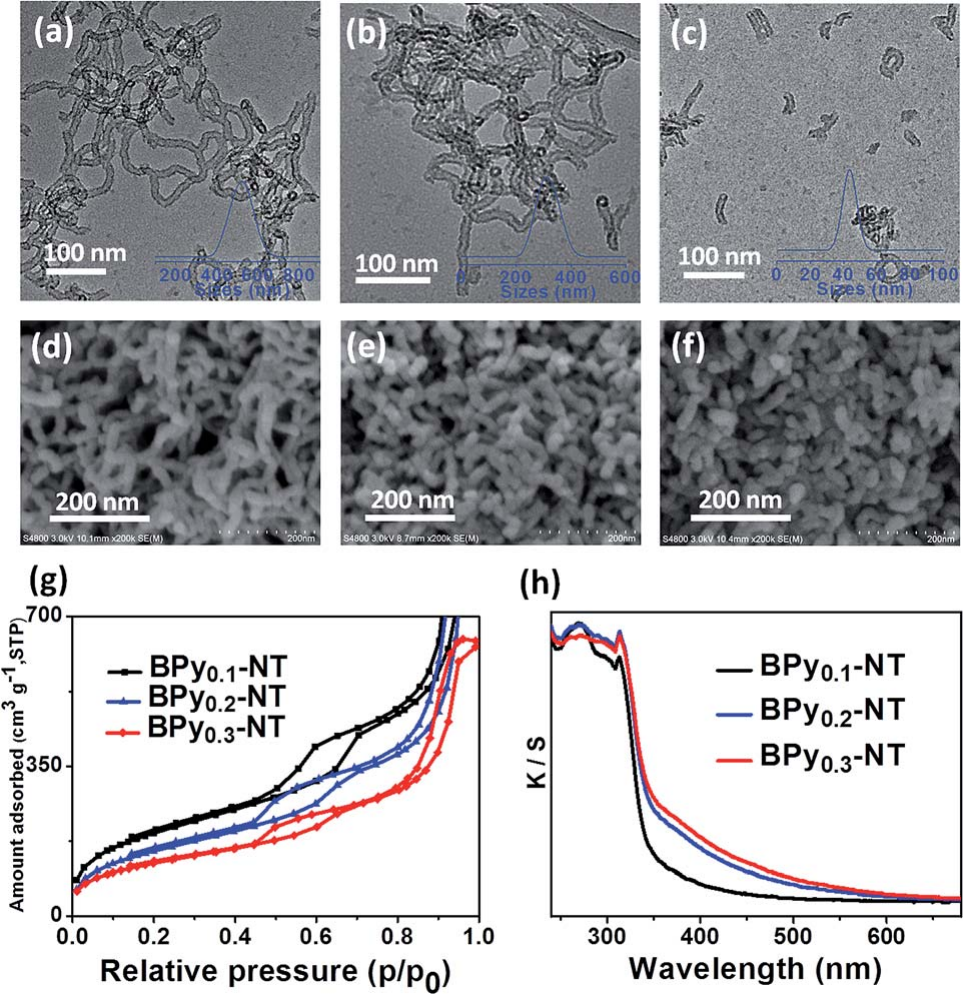
(a–f) TEM and SEM images of (a and d) BPy_0.1_-NT, (b and e) BPy_0.2_-NT, and (c and f) BPy_0.3_-NT, respectively. Insets in (a–c) are the nanotube length distribution in ethanol obtained *via* dynamics light scattering. (g) Nitrogen adsorption–desorption isotherms and (h) UV/vis diffuse reflectance spectra of BPy_0.1_-NT (black line), BPy_0.2_-NT (blue line), and BPy_0.3_-NT (red line).

Fig. 1 shows the transmission electron microscopy (TEM, a–c) and scanning electron microscopy (SEM, d–f) images of BPy_*x*_-NT with different molar ratios of bipyridine- to benzene-bridged precursors. The TEM images clearly indicated that these materials were composed of nanotubes with the inner diameter of ∼6 nm and wall thickness of ∼3 nm. The SEM images further confirmed that these nanotubes were successfully synthesized on a large scale. Note that the nanotubes could be cut short *via* adjusting the molar ratios of bipyridine- to benzene-bridged precursors during the synthetic process. When the molar ratio was 3 : 7, short nanotubes with the length of 40–60 nm were obtained (Fig. 1c and f). To date, these short organosilica nanotubes have not been successfully synthesized from bridged organosilanes.

Nitrogen adsorption–desorption isotherms of BPy_*x*_-NT are type IV with a hysteresis loop at the relative pressures *P*/*P*_0_ = 0.5–0.7, which are typical for mesoporous materials (Fig. 1g). The UV/vis spectra of BPy_*x*_-NT (Fig. 1h) displayed two main absorption peaks at around *λ* = 270 and 315 nm, corresponding to the absorption of benzene and bipyridine groups, respectively. These results indicate that mesoporous organosilica nanotubes with bipyridine ligands in the frameworks were successfully synthesized.

### Synthesis and characterization of IrCp*-BPy_*x*_-NT

**Scheme 2:**
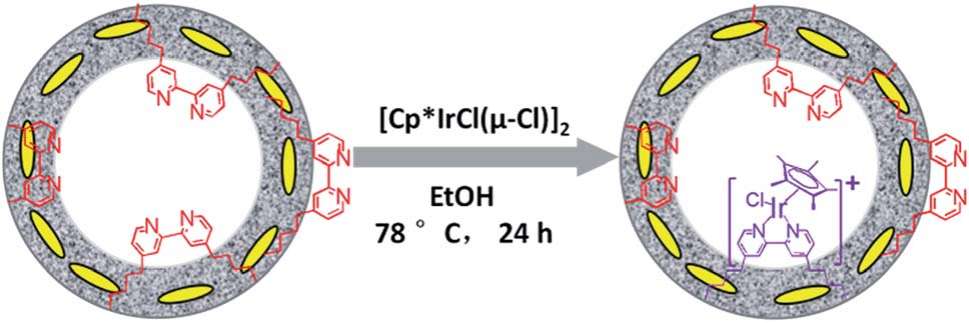
Synthetic route for the molecular heterogeneous solid catalysts IrCp*-BPy_*x*_-NT.

The direct immobilization of an iridium–Cp* complex on the nanotube walls was carried out by adding BPy_*x*_-NT to a solution of [IrCp*Cl(μ-Cl)]_2_ in anhydrous ethanol under a nitrogen atmosphere (Scheme 2), and the dried samples were named as IrCp*-BPy_*x*_-NT (*x* = 0.1, 0.2, and 0.3). Energy-dispersive X-ray (EDX) spectrum shows that the Ir loadings were 0.17, 0.18, and 0.19 mmol g^–1^ for IrCp*-BPy_0.1_-NT, IrCp*-BPy_0.2_-NT, and IrCp*-BPy_0.3_-NT, respectively, which were also confirmed by ICP measurements (Table S1†). *Via* CHN elemental analysis, the Ir/bpy molar ratios were determined to be 0.40, 0.25, and 0.17.

**Fig. 2:**
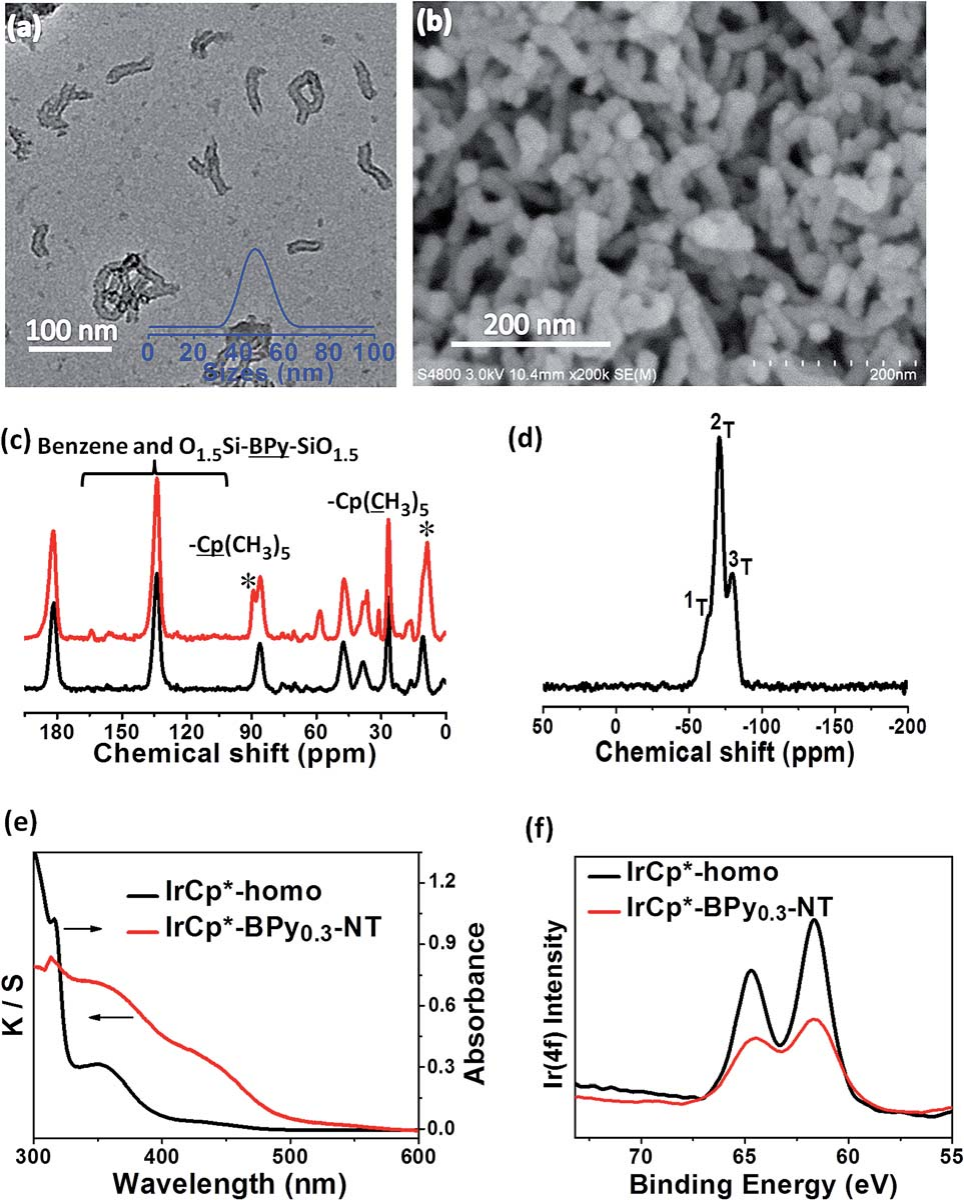
(a) TEM and (b) SEM images of IrCp*-BPy_0.3_-NT. (c) ^13^C CP MAS NMR spectra of BPy_0.3_-NT (black line) and IrCp*-BPy_0.3_-NT (red line). (d) ^29^Si MAS NMR spectrum of IrCp*-BPy_0.3_-NT. (e) UV/vis diffuse reflectance spectra and (f) XPS spectra of IrCp*-homo (black line) and IrCp*-BPy_0.3_-NT (red line).

The nanotube structure was maintained after loading the iridium–Cp* complex, as shown in Fig. 2a and b. The formation of [IrCp*Cl(bpy)]^+^ on BPy_0.3_-NT was confirmed by solid-state ^13^C cross polarization magic-angle spinning (CP MAS) NMR spectroscopy and UV/vis diffuse reflectance spectroscopy. New signals obtained at 9 and 90 ppm in the ^13^C CP MAS NMR spectra of IrCp*-BPy_0.3_-NT, compared to that of BPy_0.3_-NT, can be attributed to the Cp* ligand in IrCp*-BPy_0.3_-NT (Fig. 2c). ^29^Si MAS NMR spectrum of IrCp*-BPy_0.3_-NT (Fig. 2d) indicates intact incorporation of bipyridine groups with both ends in the framework. The UV/vis spectrum of IrCp*-BPy_0.3_-NT (Fig. 2e) displays two new peaks at around *λ* = 360 and 440 nm, similar to those obtained for homogeneous [IrCp*Cl(bpy)]Cl (denoted IrCp*-homo), which could be ascribed to a metal-to-ligand charge transfer (MLCT) transition. In addition, X-ray photoelectron spectroscopy (XPS) spectra of IrCp*-BPy_0.3_-NT is in good accordance with that of IrCp*-homo (Fig. 2f). The abovementioned characterizations indicated the successful formation of the iridium complex [IrCp*Cl(bpy)]^+^ on the organosilica nanotubes.

### Synthesis and characterization of Ir(cod)-BPy_0.3_-NT

**Scheme 3:**
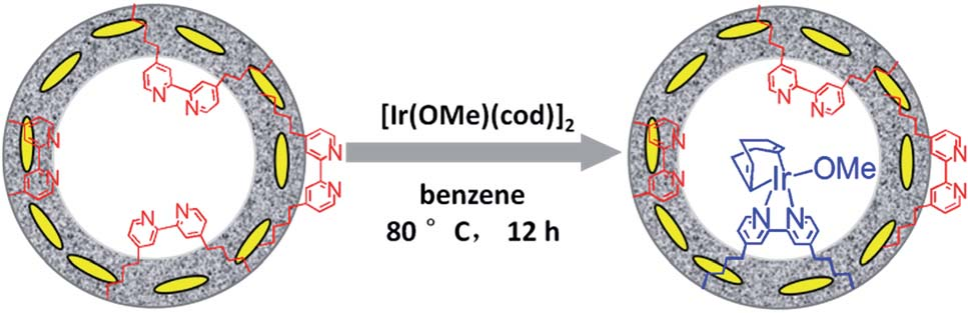
Synthetic route for the molecular heterogeneous solid catalyst Ir(cod)-BPy_0.3_-NT.

**Fig. 3:**
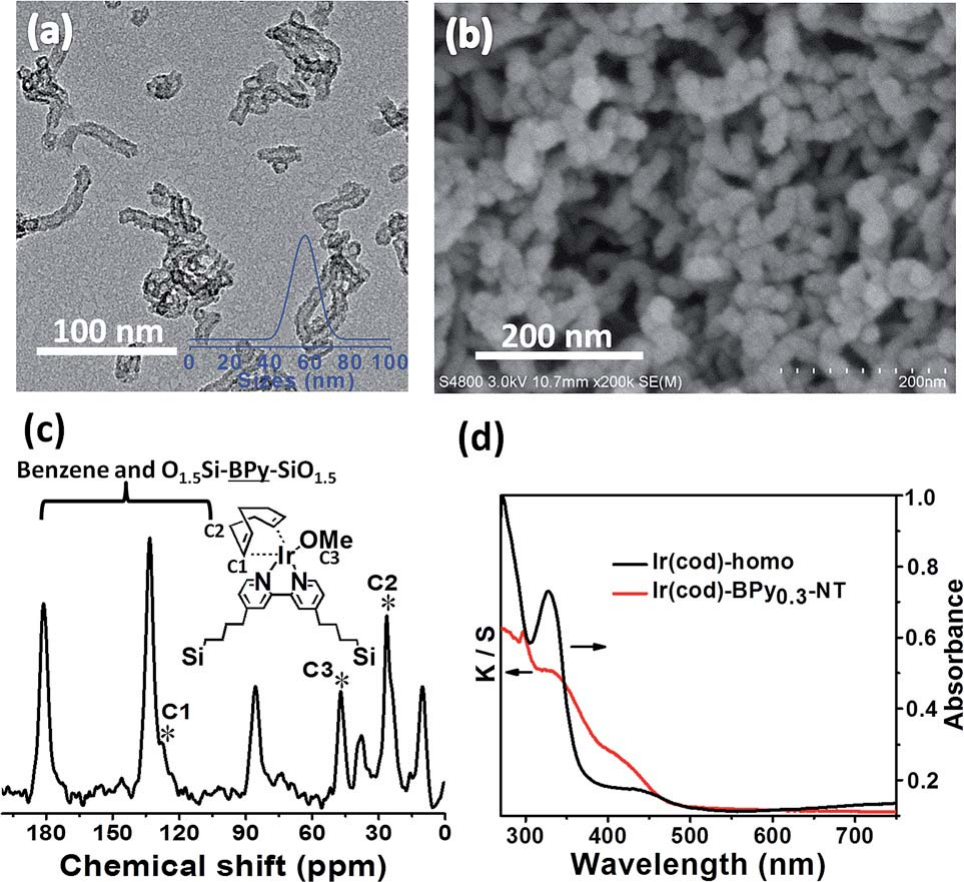
(a) TEM and (b) SEM images of Ir(cod)-BPy_0.3_-NT. (c) ^13^C CP MAS NMR spectrum of Ir(cod)-BPy_0.3_-NT. (d) UV/vis diffuse reflectance spectra of Ir(cod)-homo (black line) and Ir(cod)-BPy_0.3_-NT (red line).

Similarly, Ir(cod)-BPy_0.3_-NT was obtained by the addition of BPy_0.3_-NT to a solution of [Ir(cod)(OMe)]_2_ in 20 mL dry benzene under a nitrogen atmosphere (Scheme 3). The nanotube structure was retained after the immobilization of iridium–cod complex, as shown in Fig. 3a and b. The ^13^C CP MAS NMR spectrum (Fig. 3c), compared to that of BPy_0.3_-NT, shows new signals at 27, 48, and 128 ppm, attributed to the cod ligand in Ir(cod)-BPy_0.3_-NT. The UV/vis spectrum of Ir(cod)-BPy_0.3_-NT (Fig. 3d) exhibits two new peaks at around *λ* = 340 and 440 nm, almost similar to those of homogeneous Ir(cod)(OMe)(bpy) [denoted Ir(cod)-homo], which could be assigned to an MLCT transition. These results suggest the formation of the iridium complex Ir(cod)(OMe)(bpy) on organosilica nanotubes.

### IrCp*-BPy_*x*_-NT-catalyzed C–H oxidation

**Fig. 4:**
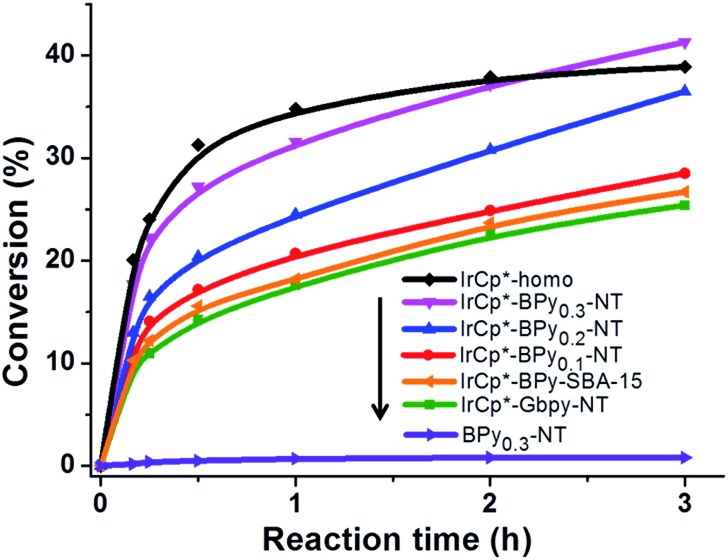
Reaction kinetic curves of THF oxidation catalyzed by IrCp*-homo (black line), IrCp*-BPy_0.3_-NT (pink line), IrCp*-BPy_0.2_-NT (blue line), IrCp*-BPy_0.1_-NT (red line), IrCp*-BPy-SBA-15 (yellow line), IrCp*-Gbpy-NT (green line), and BPy_0.3_-NT (purple line).

Catalytic performance of the as-prepared molecular heterogeneous solid IrCp*-BPy_*x*_-NT catalysts for C–H oxidation of tetrahydrofuran (THF) was investigated using NaIO_4_ as the oxidant at room temperature. According to earlier reports,^3a,b,c^ THF was first oxidized to an intermediate, 2-hydroxyl tetrahydrofuran, and then oxidized to the products butyrolactone and succinic acid. Fig. 4 illustrates the time-dependent kinetic curves of different catalysts obtained under similar reaction conditions within 3 hours. The initial TOFs based on the amounts of THF converted per unit of Ir were 1.0, 1.2, and 1.6 min^–1^ for IrCp*-BPy_0.1_-NT, IrCp*-BPy_0.2_-NT, and IrCp*-BPy_0.3_-NT, respectively (Table 1). Note that the initial TOF of IrCp*-BPy_0.3_-NT was comparable with that of the homogeneous catalyst (1.7 min^–1^) and significantly higher than those of IrCp*-BPy_0.1_-NT and IrCp*-BPy_0.2_-NT. The higher activity of IrCp*-BPy_0.3_-NT could be primarily attributed to the shorter tube length, which can reduce the diffusion limitation and facilitate the transport of reactants and products during the reactions. Furthermore, the yields of butyrolactone and succinic acid reached upto 12.8% and 9.5%, respectively, three times higher than those of IrCp*-homo (3.8% & 2.7%) within 3 hours (Table 1).

**Table 1:** IrCp*-BPy_*x*_-NT-catalyzed C–H oxidation of THF within 3 hours^*d*^

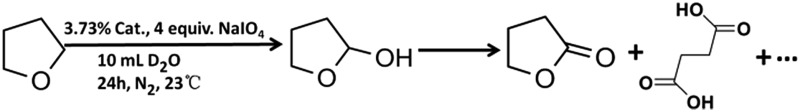				
Catalysts	TOF^*a*^/min	Conversion^*b*^/%	Yield^*c*^/%	
			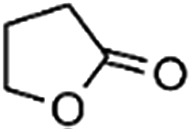	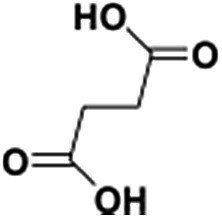
None	—	—	—	—
BPy_0.3_-NT	—	—	—	—
IrCp*-BPy_0.1_-NT	1.0	28.5	4.9	3.0
IrCp*-BPy_0.2_-NT	1.2	36.5	10.8	6.4
IrCp*-BPy_0.3_-NT	1.6	41.3	12.8	9.5
IrCp*-Gbpy-NT	0.7	25.4	2.6	2.0
IrCp*-BPy-SBA-15	0.9	26.7	4.8	4.2
IrCp*-homo	1.7	38.9	3.8	2.7

^*a*^TOF was calculated from the data within first 15 min and according to the following equation: TOF = mmol_converted THF_/(mmol_Ir_ × min).

^*b*^The conversion was calculated at 3 h.

^*c*^The yield of part products at 3 h, yield = mole of product/mole of total starting THF.

^*d*^Reaction conditions: substrate (0.6 mmol, limiting reagent), NaIO_4_ (2.4 mmol, 4 equiv.), catalysts (4.8 × 10^–3^ mmol Ir), D_2_O (10 mL), at room temperature under N_2_.

**Scheme 4:**
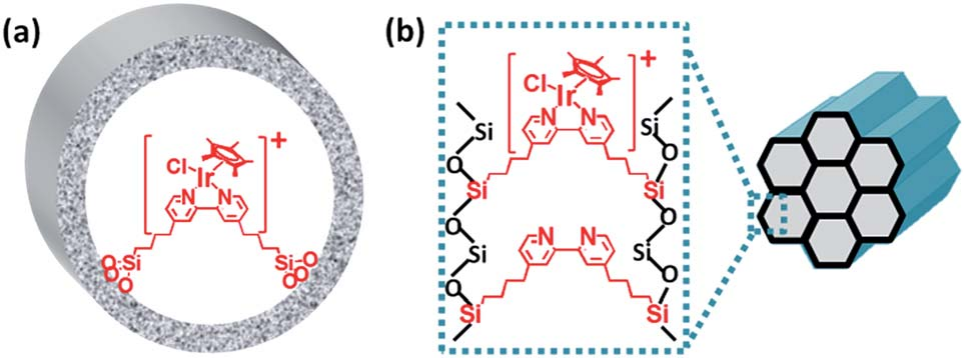
Schematic of (a) IrCp*-Gbpy-NT and (b) IrCp*-BPy-SBA-15.

For comparison, [IrCp*Cl(bpy)]^+^ complex was immobilized on bipyridine-grafted nanotubes through a grafting method (Scheme 4a and Fig. S10–13†). However, the grafted iridium complexes IrCp*-Gbpy-NT exhibited lower TOF (0.7 min^–1^) and yield than IrCp*-BPy_*x*_-NT (Table 1). The lower activity of IrCp*-Gbpy-NT can be attributed to the non uniformity of the catalytic sites in the nanotubes and undesirable interactions of the metal Ir active center due to the protrusion of iridium complexes into the nanotube channels. We also examined the heterogenization of the homogeneous [IrCp*Cl(bpy)]Cl complex on the conventional mesoporous support benzene-bridged mesoporous organosilicas (B-SBA-15) (Scheme 4b and Fig. S14–18†). This heterogeneous catalyst exhibited lower TOF (0.9 min^–1^) than IrCp*-BPy_0.3_-NT, possibly because of the diffusion effects in SBA-15. The abovementioned results demonstrate that the novel BPy-NT can effectively reduce the diffusion limitation and facilitate the transport of reactants and products during the reactions due to the uniform short nanotube structure and the large pore diameter.

**Fig. 5:**
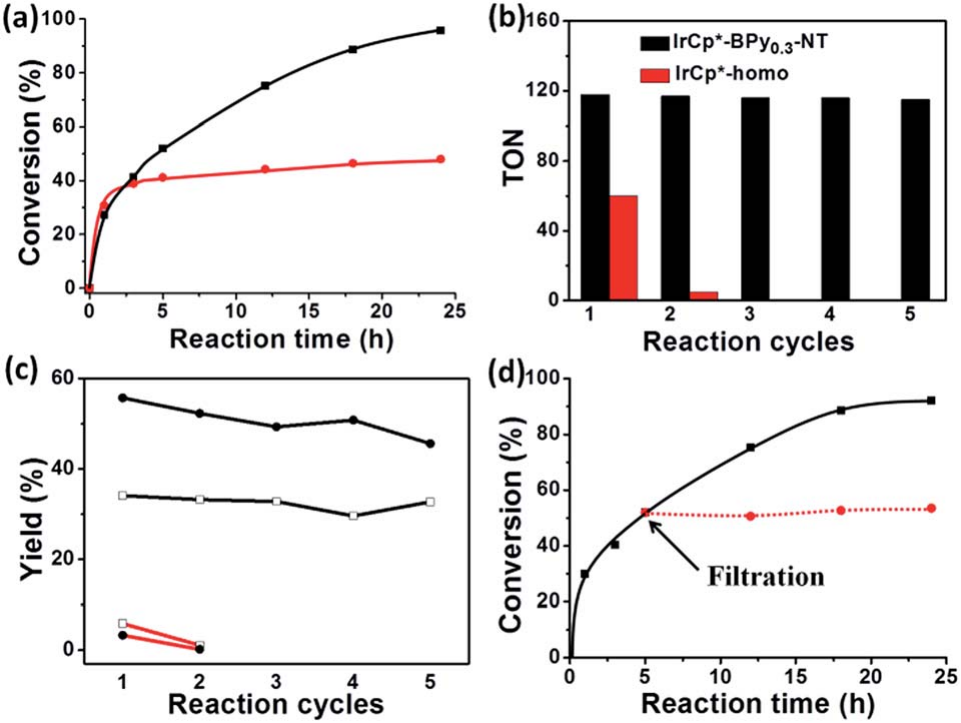
(a) Reaction kinetic curves of THF oxidation catalyzed by IrCp*-BPy_0.3_-NT (black line) and IrCp*-homo (red line) within 24 h. (b) Reusability of IrCp*-BPy_0.3_-NT and IrCp*-homo catalysts. (c) The yields of products (□: butyrolactone, : succinic acid) for IrCp*-BPy_0.3_-NT (black line) and IrCp*-homo (red line) catalyst for reaction recycles. (d) Filtration experiment of IrCp*-BPy_0.3_-NT catalyst.

By further extending the reaction time to 24 h (Fig. 5a and entry 1 in Table 2), the conversion of THF reached up to 95.8% with the yields of 34.1% of butyrolactone and 55.7% of succinic acid for IrCp*-BPy_0.3_-NT. Moreover, IrCp*-homo presented only 48% conversion with the yields of 5.9% of butyrolactone and 3.3% of succinic acid. The mass spectrum (MS) (Fig. S35†) revealed that the peak of IrCp*-homo molecule at 519 disappeared after the reaction, indicating the total decomposition of the homogeneous catalyst into inactive species, which caused the deactivation of IrCp*-homo.^3a,b^ On contrary, IrCp*-BPy_0.3_-NT remained active and more amounts of products were obtained. BPy-NT has the potential of suppressing unfavourable interactions and aggregation of Ir active centers due to isolated binding of metals on well-defined surface.

**Table 2:** IrCp*-BPy_*x*_-NT-catalyzed C–H oxidation of different substrates^*d*^

Entry	Substrate	Products	TOF^*a*^/min	Conversion^*b*^/%	Yield^*c*^/%
1	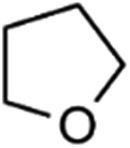	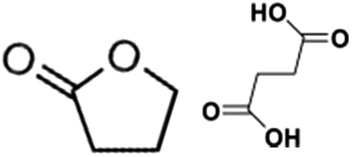	1.6	95.8	34.1/55.7
2	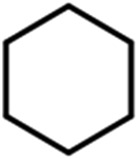	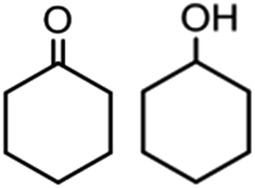	1.4	99.7	20.1/21.3
3	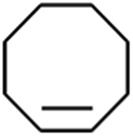	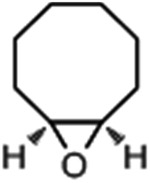	4.3	93.4	58.8
4	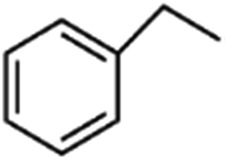	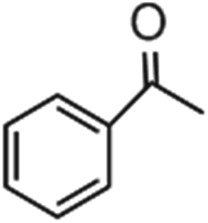	1.2	24.7	6.4
5	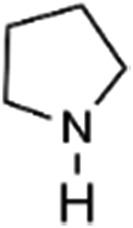	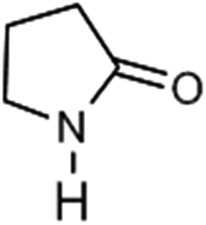	1.0	11.3	3.0

^*a*^TOF was calculated from the data within first 15 min and according to the following equation: TOF = mmol_converted substrate_/(mmol_Ir_ × min).

^*b*^The conversion was obtained at 24 h.

^*c*^Yield = mole of product/mole of total starting material.

^*d*^Reaction conditions: substrate (0.6 mmol, limiting reagent), NaIO_4_ (2.4 mmol, 4 equiv.), catalysts (4.8 × 10^–3^ mmol Ir), at room temperature under N_2_.

The reusability of IrCp*-BPy_0.3_-NT was investigated at 24 h intervals. After the reaction, IrCp*-BPy_0.3_-NT was reused for the next run and the solid catalyst retained high catalytic activity (Fig. 5b and c). For the 5^th^ recycle, TON was still retained at around 115 with the high yields of 32.7% of butyrolactone and 45.6% of succinic acid. After recycling, the solid catalyst was removed from the reaction and the solid-free solution obtained was colorless (Fig. S36a†), indicating that the Ir complex was firmly coordinated with BPy-NT without leaching of Ir species, which was also confirmed by ICP analysis. In addition, the filtration experiment was conducted to examine if the solid catalyst was truly heterogeneous (Fig. 5d). After stirring the reaction system for 5 h, a conversion of 52% was achieved and the solid catalyst was filtered off from the reaction mixture under a nitrogen atmosphere; the remaining solution was stirred for another 19 h. The conversion of THF had no prominent increase, thereby indicating that the reaction completely ceased and the catalytic activity indeed originated from IrCp*-BPy_0.3_-NT. These results suggest that IrCp*-BPy_0.3_-NT has high stability and no leaching of Ir species occurred. In contrast, the homogeneous catalyst showed almost no catalytic performance after recycling (Fig. 5b).

To further understand the origin of the active sites in IrCp*-BPy_0.3_-NT, the recovered catalyst was characterized by TEM, SEM, nitrogen adsorption, UV-vis, XPS, and solid-state NMR spectroscopy (Fig. S36–42†). TEM and SEM images show the intact nanotube structure even after the 5^th^ recycle, indicating that the support was stable for C–H oxidation (Fig. S36b and c†). The UV/vis spectrum of IrCp*-BPy_0.3_-NT after the reaction shows two peaks at around *λ* = 360 and 440 nm originating from the [IrCp*Cl(bpy)]^+^ complex, which could testify the firm coordination of Ir with bipyridine ligands (Fig. S38†). XPS data revealed that the valence state of Ir after the reaction was III (Fig. S39†). The ^13^C CP MAS NMR spectrum shows a gradual decrease in the signals of the Cp* rings (9 and 90 ppm) with an increase in number of recycle times because of the oxidative decomposition (Fig. S40†), which was also found during the water oxidation reaction catalyzed by [IrCp*Cl(bpy)]^+^ complex.^23^ The EDX analysis results show uniform distribution of iridium on BPy-NT, suggesting no formation of iridium oxide particles (Fig. S41†). Furthermore, the framework composed of bipyridine and benzene was quite stable during the reaction, as observed from the ^29^Si MAS NMR spectrum (Fig. S42†).

To verify the universality, the nanotube catalytic system was examined using different substrates such as cyclohexane, ethylbenzene, pyrrolidine, and cyclooctene (entry 2–5 in Table 2). Particularly, IrCp*-BPy_0.3_-NT could catalyze cyclohexane activation with a high conversion of 99.7%. The yields for cyclohexanone and cyclohexanol were attained as 20.1% and 21.3%, respectively (entry 2 in Table 2). Cyclooctene epoxidation to cyclooctene oxide with a high conversion of 93.4% and yield of 58.8% was observed by IrCp*-BPy_0.3_-NT (entry 3 in Table 2). Moreover, pyrrolidine could be oxidized to 2-pyrrolidinone with 11.3% conversion and 3.0% yield (entry 5 in Table 2), while IrCp*-BPy_0.3_-NT did not show any activity in the homogeneous system.^3*c*^

### Ir(cod)-BPy_0.3_-NT-catalyzed C–H borylation of arenes

**Fig. 6:**
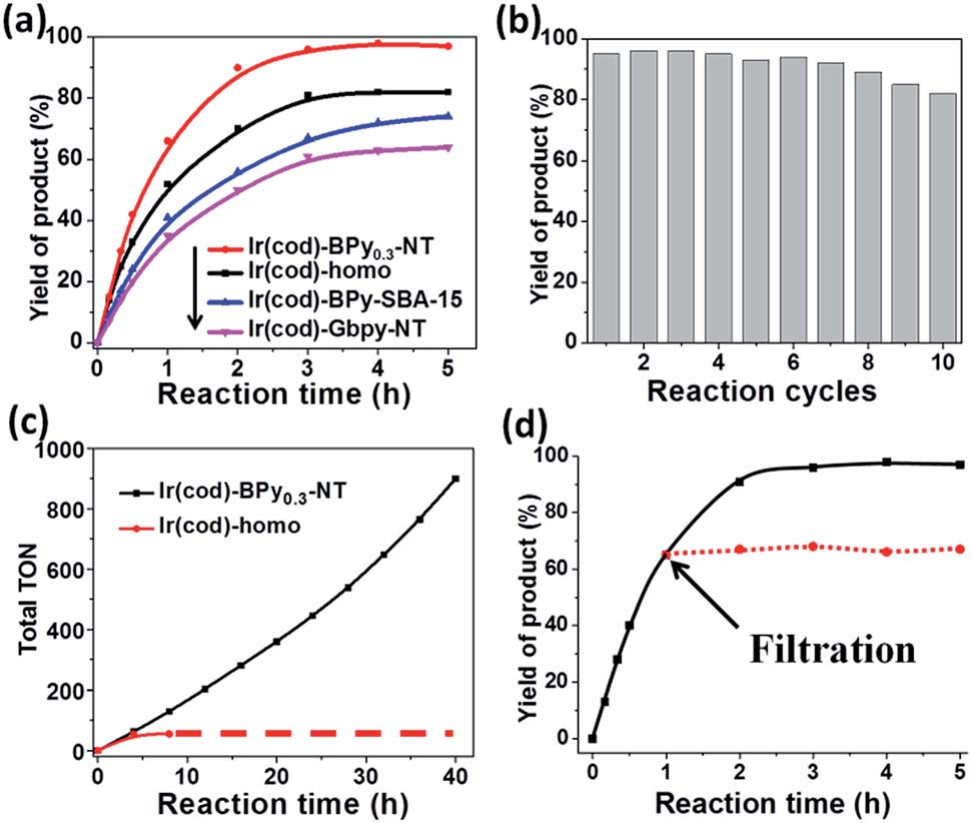
(a) Reaction kinetic curves of benzene borylation catalyzed by Ir(cod)-homo (black line), Ir(cod)-BPy_0.3_-NT (red line), Ir(cod)-BPy-SBA-15 (blue line), and Ir(cod)-Gbpy-NT (pink line). (b) Reusability of Ir(cod)-BPy_0.3_-NT for benzene borylation. (c) Total TONs of Ir(cod)-BPy_0.3_-NT (black line) and Ir(cod)-homo (red line) for the recycling. (d) Filtration experiment of Ir(cod)-BPy_0.3_-NT catalyst (red-dotted line).

Direct C–H borylation of arenes by molecular heterogeneous Ir(cod)-BPy_0.3_-NT catalyst was performed using B_2_(pin)_2_ (pin = pinacolate) as a boron source at 80 °C. Within 12 h, Ir(cod)-BPy_0.3_-NT gave a high yield of 97% for benzene borylation, which was significantly higher than those obtained using the homogeneous catalyst Ir(cod)-homo (82%), grafted catalyst on organosilica nanotubes Ir(cod)-Gbpy-NT (68%) (Fig. S22–25 and Scheme S1†), and incorporated catalyst on mesoporous organosilicas Ir(cod)-BPy-SBA-15 (80%) (Fig. S26–30 and Scheme S2†) (entry 1–4 in Table 3). Fig. 6a shows the reaction kinetics for directed benzene borylation using different catalysts. Ir(cod)-BPy_0.3_-NT exhibited high initial reactivity similar to that of the homogeneous catalyst, indicating little diffusion limitation in the short nanotube channels. Furthermore, Ir(cod)-BPy_0.3_-NT performed constant high activity during the reaction until B_2_(pin)_2_ was used up, whereas the reaction catalyzed by the homogeneous catalyst and grafted Ir(cod)-Gbpy-NT almost ceased after 3 h due to the deactivation of the catalysts.^21^ The incorporated catalyst Ir(cod)-BPy-SBA-15 showed lower reaction rates than Ir(cod)-BPy_0.3_-NT. This could be due to the diffusion limitation in SBA-15. These results further demonstrate the advantages of BPy_0.3_-NT due to the isolated active sites as well as the fast transport in the nanotube channel.

**Table 3:** Ir(cod)-BPy_0.3_-NT-catalyzed C–H borylation of arenes^*c*^

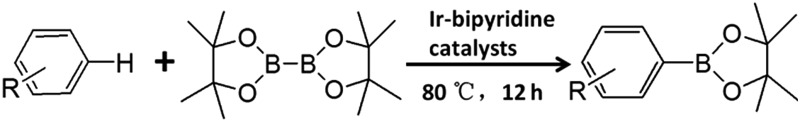				
Entry	R	Catalysts	Yield^*a*^/%	TON^*b*^
1	H	Ir(cod)-BPy_0.3_-NT	97	64
2	H	Ir(cod)-homo	82	54
3	H	Ir(cod)-Gbpy-NT	68	45
4	H	Ir(cod)-BPy-SBA-15	80	53
5	CH_3_	Ir(cod)-BPy_0.3_-NT	90	59
6	1,2-(CH_3_)_2_	Ir(cod)-BPy_0.3_-NT	81	53
7	1,3-(CH_3_)_2_	Ir(cod)-BPy_0.3_-NT	86	57
8	OMe	Ir(cod)-BPy_0.3_-NT	94	62
9	1,2-(OMe)_2_	Ir(cod)-BPy_0.3_-NT	95	63
10	1,2-Cl_2_	Ir(cod)-BPy_0.3_-NT	97	64
11	1,4-Cl_2_	Ir(cod)-BPy_0.3_-NT	92	61

^*a*^The average ^1^H NMR yields of aryl boronate based on the protons of the Bpin group in the product and in B_2_pin_2_.

^*b*^TON = mole of product/mole of Ir.

^*c*^Reaction conditions: arenes (20 mmol), B_2_pin_2_ (0.33 mmol, limiting reagent), catalysts (5.0 × 10^–3^ mmol Ir), at 80 °C, 12 h under N_2_.

The reusability of Ir(cod)-BPy_0.3_-NT for benzene borylation was examined at 4 h intervals (Fig. 6b). The recovered solid catalyst still exhibited high catalytic activity (82% yield) with a slight loss in the product yield after the 10^th^ recycle, which was due to the unavoidable catalyst loss during the filtration and washing process. However, the homogeneous catalyst showed almost no reaction activity for the recycling because of deactivation.^21^ The total TONs for Ir(cod)-BPy_0.3_-NT after the 10^th^ reuse could reach upto 900, 16 times higher than that of the homogeneous catalyst (54, Fig. 6c). The heterogeneity of Ir(cod)-BPy_0.3_-NT was also confirmed by the filtration experiment (Fig. 6d). The analysis of the recovered catalysts by TEM, SEM, and nitrogen adsorption (Fig. S43 and S44†) indicated that the nanotube structure was intact during the reactions. UV-vis and ^13^C CP MAS NMR (Fig. S45 and S46†) show the almost intact structure of the active site in Ir(cod)(OMe)(bpy). The uniform distribution of iridium analyzed by EDX indicates no aggregation of molecular catalysts (Fig. S47†). ^29^Si MAS NMR spectrum shows no formation of Q sites, thereby suggesting that the framework composition was stable even after the 10^th^ recycle (Fig. S48†).

The nanotube catalytic system also exhibited high catalytic activities for C–H borylation of various types of benzene derivatives (entry 5–11 in Table 3). Note that Ir(cod)-BPy_0.3_-NT could effectively catalyze C–H borylation of substrates with large molecular sizes to high yields in less reaction time, when compared to MOFs,^19*b*^ because of the large pore diameter (5 nm) of the nanotubes than that of the MOFs (*ca.* 1 nm).

## Conclusions

In conclusion, the original organosilica nanotubes containing iridium–bipyridine complexes IrCp*-BPy-NT or Ir(cod)-BPy-NT were obtained respectively by the post-synthetic metalation of BPy-NT with Ir complex precursors [IrCp*Cl(μ-Cl)]_2_ or [Ir(cod)(OMe)]_2_. The molecular heterogeneous Ir-based catalysts were successfully applied in the heterogeneous catalytic C–H oxidation and C–H borylation reactions, which showed high catalytic activity and durability owing to the effective suppression of iridium–bipyridine complex aggregation as well as fast transport in short nanotubes. The characterizations for Ir-BPy_*x*_-NT after the reactions indicated that the active species had a molecular structure, not as that of iridium oxide or iridium nanoparticles. These results demonstrate the potential of BPy-NT with a short length as a heterogeneous solid support and an integration platform for the heterogeneous catalysis systems of organic transformations.

## References

[cit1] Huang Z., Lim H. N., Mo F., Young M. C., Dong G. (2015). Chem. Soc. Rev..

[cit2] Howell J. M., Feng K., Clark J. R., Trzepkowski L. J., White M. C. (2015). J. Am. Chem. Soc..

[cit3] Zhou M., Hintermair U., Hashiguchi B. G., Parent A. R., Hashmi S. M., Elimelech M., Periana R. A., Brudvig G. W., Crabtree R. H. (2013). Organometallics.

[cit4] Zhou M., Crabtree R. H. (2011). Chem. Soc. Rev..

[cit5] Liu W., Yu Q., Hu L. a., Chen Z., Huang J. (2015). Chem. Sci..

[cit6] Cui Y., He C. (2004). Angew. Chem., Int. Ed..

[cit7] Gong J. L., Yue H. R., Zhao Y. J., Zhao S., Zhao L., Lv J., Wang S. P., Ma X. B. (2012). J. Am. Chem. Soc..

[cit8] Copéret C., Estes D. P., Larmier K., Searles K. (2016). Chem. Rev..

[cit9] Conley M. P., Copéret C., Thieuleux C. (2014). ACS Catal..

[cit10] Li B., Li F., Bai S. Y., Wang Z. J., Sun L. C., Yang Q. H., Li C. (2012). Energy Environ. Sci..

[cit11] Wu F., Feng Y., Jones C. W. (2014). ACS Catal..

[cit12] Karakhanov E., Maximov A., Kardasheva Y., Semernina V., Zolotukhina A., Ivanov A., Abbott G., Rosenberg E., Vinokurov V. (2014). ACS Appl. Mater. Interfaces.

[cit13] Copéret C., Basset J. M. (2007). Adv. Synth. Catal..

[cit14] Estes D. P., Siddiqi G., Allouche F., Kovtunov K. V., Safonova O. E., Trigub A. L., Koptyug I. V., Coperet C. (2016). J. Am. Chem. Soc..

[cit15] Noh H., Cui Y., Peters A. W., Pahls D. R., Ortuno M. A., Vermeulen N. A., Cramer C. J., Gagliardi L., Hupp J. T., Farha O. K. (2016). J. Am. Chem. Soc..

[cit16] Genna D. T., Pfund L. Y., Samblanet D. C., Wong-Foy A. G., Matzger A. J., Sanford M. S. (2016). ACS Catal..

[cit17] Maschmeyer T., Rey F., Sankar G., Thomas J. M. (1995). Nature.

[cit18] Tagata T., Nishida M., Nishida A. (2009). Tetrahedron Lett..

[cit19] Wang C., Xie Z., deKrafft K. E., Lin W. (2011). J. Am. Chem. Soc..

[cit20] Maegawa Y., Inagaki S. (2015). Dalton Trans..

[cit21] Waki M., Maegawa Y., Hara K., Goto Y., Shirai S., Yamada Y., Mizoshita N., Tani T., Chun W. J., Muratsugu S., Tada M., Fukuoka A., Inagaki S. (2014). J. Am. Chem. Soc..

[cit22] Grüning W. R., Siddiqi G., Safonova O. V., Copéret C. (2014). Adv. Synth. Catal..

[cit23] Liu X., Maegawa Y., Goto Y., Hara K., Inagaki S. (2016). Angew. Chem., Int. Ed..

[cit24] Liu X., Li X., Guan Z., Liu J., Zhao J., Yang Y., Yang Q. (2011). Chem. Commun..

[cit25] Fischer C. E., Raith A., Mink J., Raudaschl-Sieber G., Cokoja M., Kühn F. E. J. (2011). Organomet. Chem..

[cit26] Zhang X., Su F., Song D., An S., Lu B., Guo Y. (2015). Appl. Catal., B.

[cit27] Song D., An S., Sun Y., Guo Y. (2016). J. Catal..

[cit28] Nicewicz D. A., MacMillan D. W. C. (2008). Science.

[cit29] Concepcion J. J., Jurss J. W., Brennaman M. K., Hoertz P. G., Patrocinio A. O. T., Iha N. Y. M., Templeton J. L., Meyer T. J. (2009). Acc. Chem. Res..

[cit30] Yin Q. S., Tan J. M., Besson C., Geletii Y. V., Musaev D. G., Kuznetsov A. E., Luo Z., Hardcastle K. I., Hill C. L. (2010). Science.

