# Supplementary material for: Molecular heterogeneous catalysts derived from bipyridine-based organosilica nanotubes for C–H bond activation
Source: Chem Sci. 2017 Apr 19;8(6):4489–96. doi: 10.1039/c7sc00713b (PMC5618254; doi:10.1039/c7sc00713b)
Supplement: Supplementary file 1 [file SC-008-C7SC00713B-s001.pdf]

Electronic Supplementary Material (ESI) for Chemical Science.
This journal is © The Royal Society of Chemistry 2017

Supporting Information for

Molecular Heterogeneous Catalysts Derived from Bipyridine-based Organosilica Nanotubes for C-H Bond Activation

Shengbo Zhang, Hua Wang, Mei Li, Jinyu Han, Xiao Liu*, Jinlong Gong*

Key Laboratory for Green Chemical Technology, School of Chemical Engineering and Technology, Tianjin University; Collaborative Innovation Center of Chemical Science and Engineering, Tianjin 300072, China

*Email: liuxiao71@tju.edu.cn; jlgong@tju.edu.cn

Table of Contents

1. Chemicals

2. Characterization

3. Synthesis of 4,4'-[4-(Trimethoxysilanyl)butyl]-2,2'-bipyridine (1).

4. Synthesis of BPy-NT and IrCp*-BPy-NT

5. Synthesis of IrCp*-Gbpy-NT

6. Synthesis of IrCp*-BPy-SBA-15

7. Synthesis of Ir(cod)-BPy$_{0.3}$-NT

8. Synthesis of Ir(cod)-Gbpy-NT

9. Synthesis of Ir(cod)-BPy-SBA-15

10. Ir-BPy-NT-catalyzed C-H activation reaction

11. Reusability test for C-H activation reaction

12. The analysis of homogeneous catalyst by using mass spectrum

13. The structure analysis of Ir-BPy$_{0.3}$-NT after the reaction

14. References

1. Chemicals

4,4′-dimethyl-2,2′-dipyridyl (98%), *n*-butyllithium (*n*-BuLi; 1.6 M in hexane), tetrahydrofuran (THF; ＞99%), triblock copolymer $EO_{20}PO_{70}EO_{20}$ (Pluronic P123, M_w = 5800), 1,4-bis(triethoxysilyl)benzene (BTEB) and 2,2′-bipyridine were purchased from Sigma-Aldrich Company Ltd. (U.S.A.). Diisopropylamine (DiPA; ＞99.5%), $NaIO_4$ (＞99.5%), pentamethylcyclopentadienyliridium(III) chloride, dimer ($[Cp^*IrCl_2]_2$, Cp* is the pentamethylcyclopentadienyl) (96%), $[Ir(OMe)(cod)]_2$ (96%), and bis(pinacolato)diboron (B_2pin_2) (98%) were obtained from Aladdin. (3-chloropropyl)-trimethoxylsilane was received from Heowns (95%). Other reagents were obtained from Shanghai Chemical Reagent. All solvents were of analytical quality and dried by standard methods.

[IrCp*Cl(bpy)]Cl and Ir(cod)(OMe)(bpy) were prepared as previous reports[1,2], which were denoted as IrCp*-homo and Ir(cod)-homo, respectively.

2. Characterization

Transmission electron microscopys (TEM) were operated on a JEM-2100F system operating at 200 kV. The morphology of these catalysts were analysed by scanning electron microscopy (SEM) using Hitachi S-4800 scanning electron microscope (SEM, 5 kV) equipped with the Thermo Scientific energy-dispersion X-ray fluorescence analyser. N_2 adsorption-desorption isotherms were tested at -196 ºC on a Micromeritics Tristar 3000 instrument. The specific surface area was obtained by the Brunauer–Emmett–Teller method; the pore size distribution was obtained by the Barrett–Joyner–Halenda method using the adsorption branch. UV/vis absorption and

diffuse reflectance spectra were obtained using Instant Spec BWS003 spectrometers. FT-IR spectra were collected with a Bruker Vertex 80v. Carbon, hydrogen and nitrogen contents were determined via CHN elemental analysis. Solid-state ^{13}C cross polarization magic-angle spinning (CP MAS) NMR and ^{29}Si magic angle spinning (MAS) NMR spectra were collected on 400 MHz instruments. Solution ^{1}H NMR and ^{13}C NMR spectra were recorded on a Mercury VX 400 MHz instrument.

3. Synthesis of 4,4′-[4-(Trimethoxysilanyl)butyl]-2,2′-bipyridine (1).

The synthesis procedure is as follows: In a 250 mL flask, 1.476 g diisopropylamine and 40 mL anhydrous THF were added under nitrogen; 8.48 mL n-BuLi (1.6 M, 13.2 mmol) was added dropwise to this solution under ice-water bath. After the mixture solution was stirred for 30 min, a solution of 2.42 g 4,4’-dimethyl-2,2’-bipyridine (13.2 mmol) in 100 mL anhydrous THF was added slowly. The mixture solution was stirred for an additional 1 h at 0 ºC under nitrogen. Then, a solution of 5.24 g (3-chloropropyl)trimethoxylsilane (4.82 mL, 29 mmol) in 10 mL anhydrous THF was added slowly. After the solution was stirred 2 h, the reaction mixture was allowed to increase to room temperature and then stirred overnight before quenching with three drops of acetone. The remaining solvent and (3-chloropropyl)trimethoxylsilane were removed by vacuum distillation to obtain the product, alkoxysilane-modified 2, 2′-bipyridine as a pale yellow solid **(1)** (yield 4.70 g, 68%). Finally, the product is stored in 33 mL anhydrous THF. ^{1}H NMR (DMSO, 400 MHz): δ= 8.48 (m, 2H, Py), 8.24 (s, 2H, Py), 7.19 (d, J=6.8 Hz, 2H, Py), 3.58 (s, 18H, OMe), 2.39 (t, J=6.4 Hz, 4H, CH_2-Py), 1.73 (m, 4H, CH_2), 1.25 (m, 4H, CH_2), 0.60 (t, J=0.8 Hz, 4H,

CH_2-Si).^{13}C NMR (DMSO, 400 MHz): δ= 10.6, 14.9, 35.6, 35.8, 42.8, 121.4, 124.8, 147.9, 149.2, 155.7. ESI-MS: *m/z* calcd. for $C_{24}H_{40}N_2O_6Si_2$: 508.7552; found: 508.6950.

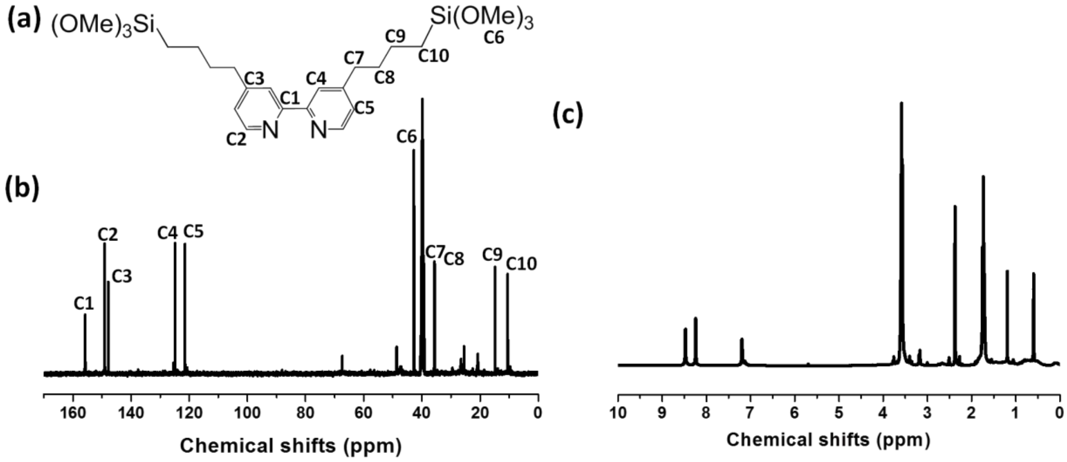

Figure S1. (a)The structure of bipyridine- precursor **(1)**, (b) ^{13}C NMR spectrum and (c) ^{1}H NMR spectrum of bipyridine- precursor **(1)** in *d*-DMSO.

4. Synthesis of BPy-NT and IrCp*-BPy-NT

The method to synthesize organosilica nanotubes was according to our previous report with minor modifications.[3] In a typical synthesis, 0.55 g of P123 and 1.75 g of KCl were dissolved in 135 ml 2 M HCl solution at 38 °C. After the copolymer was fully dissolved, 2.45 mmol of 1, 4-bis(triethoxysilyl)benzene (BTEB) **(2)** was added with vigorous stirring for 6 min. After stirring for 12 h at 38 °C, 1.05 mmol of **(1)** dissolved in 2.7 ml THF was added dropwise. The molar ratio of bipyridine-bridged precursor **(1)** to BTEB **(2)** in the resulting mixture was 3:7. Then the mixture was stirred at 38 °C for 24 h and the resultant mixture was transferred to a PTFE hydrothermal reactor at 100 °C for an additional 24 h. The solid product was recovered by filtration and was dried at room temperature overnight. Finally, the surfactant was extracted by refluxing 1.0 g of the as-synthesized material in 200 ml of ethanol containing 1.27 g concentrated HCl for 24 h. The obtained sample was denoted $BPy_{0.3}$-NT. By contrast, the nanotubes with molar ratio of bipyridine-bridged precursor **(1)** to BTEB (2) 1:9 and 2:8 were also synthesized.

The iridium-immobilized nanotube catalysts were prepared by adding BPy-NT (100 mg) to a solution of $[Cp^*IrCl(\mu\text{-}Cl)]_2$ (10 mg) in 60 ml anhydrous ethanol under an nitrogen atmosphere. After the suspension was stirred under refluxing conditions for 24 h, the solid phase was obtained by filtration and washed with DMF (*N*,*N*-dimethylformamide) and distilled water to remove unreacted $[Cp^*IrCl(\mu\text{-}Cl)]_2$. The dried samples were named as IrCp*-BPy_x-NT, where x = 0.1, 0.2 and 0.3,

respectively, responding to the molar ratio of bipyridine- to benzene-bridged precursor in the initial synthesis of BPy-NT.

TEM and SEM analysis:

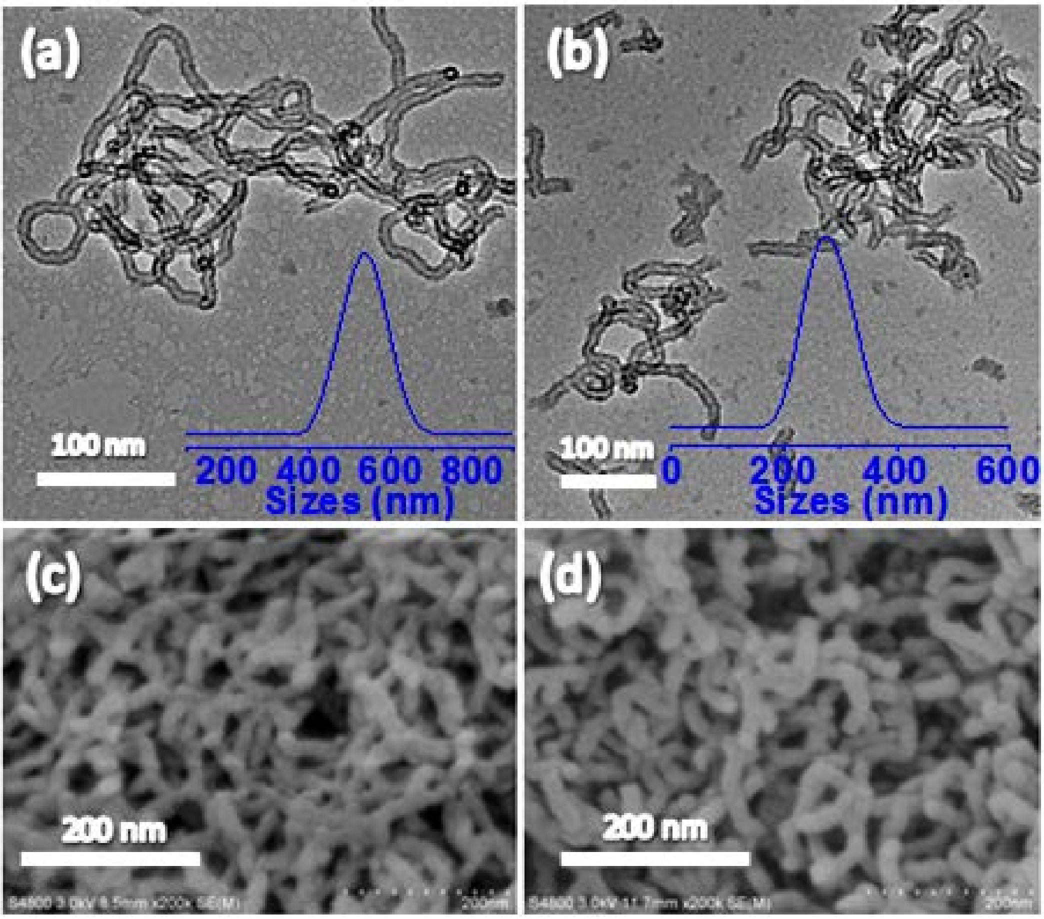

Figure S2. The TEM and SEM images of (a,c) IrCp*-$BPy_{0.1}$-NT, (b,d) IrCp*-$BPy_{0.2}$-NT.

Elemental analysis

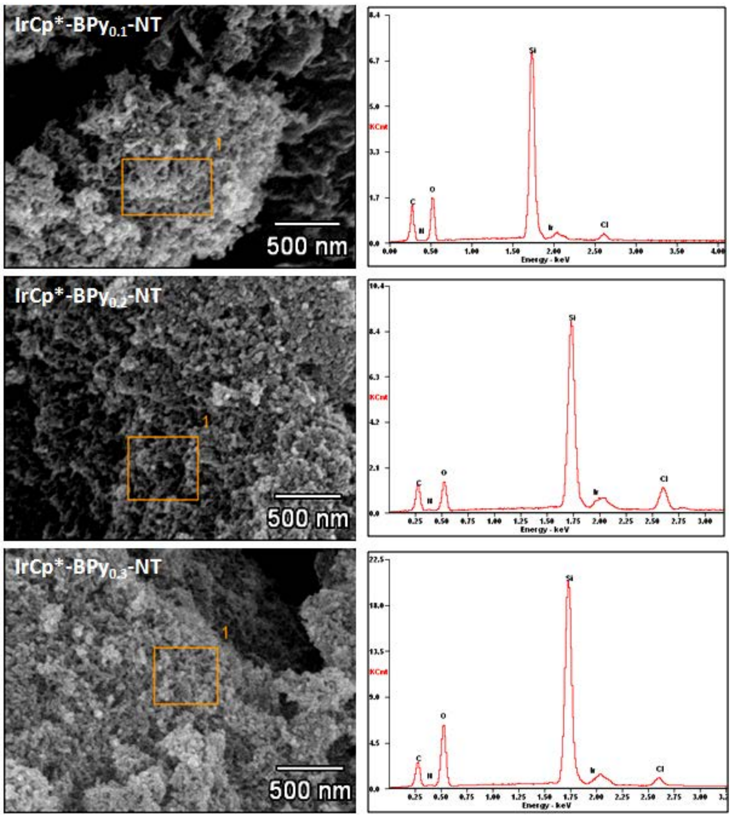

Figure S3. EDX analysis of IrCp*-BPy$_x$-NT.

Table S1. Elemental analysis results of IrCp*-BPy-NT

Materials	C%	H%	[a]N%	[b]Si%	[c]Ir%	[d]Ir%	Ir (mmol/g)
IrCp*-BPy$_{0.1}$-NT	38.73	4.7	1.20	33.10	3.25	3.43	0.17
IrCp*-BPy$_{0.2}$-NT	39.50	4.0	2.13	31.76	3.64	3.71	0.18
IrCp*-BPy$_{0.3}$-NT	38.72	4.3	3.12	30.09	3.73	3.76	0.19

[a]The nitrogen content was based on CHN elemental analysis. [b]The values of Si were measured by energy-dispersive X-ray spectroscopy (EDX) instrumentation attached to the SEM system (Hitachi S-4800). [c]The values of Ir loadings were measured by EDX. [d]The values of Ir loadings were measured by ICP analysis.

Ir distribution analysis:

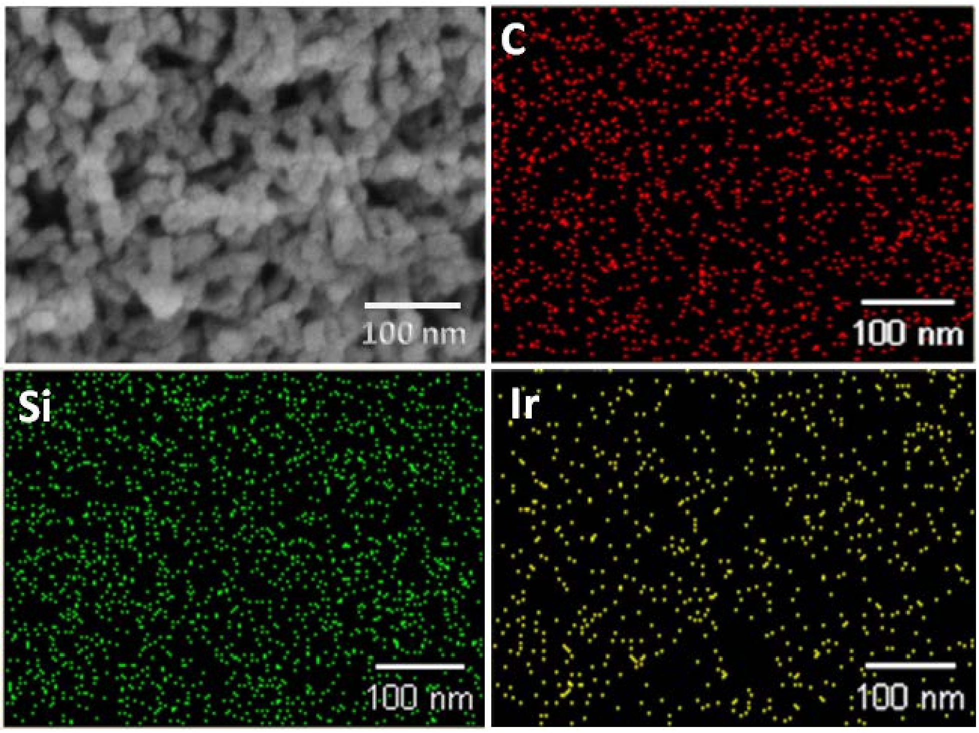

Figure S4. EDX analysis of IrCp*-BPy$_{0.3}$-NT.

Figure S4 shows the mapping analysis of the chosen area by energy-dispersive X-ray spectrometry (EDX) with the scanning electron microscopy (SEM) using Hitachi S-4800 (5 kV) equipped with the Thermo Scientific energy-dispersion X-ray fluorescence analyzer, which indicated that Ir was uniformly distributed on the support.

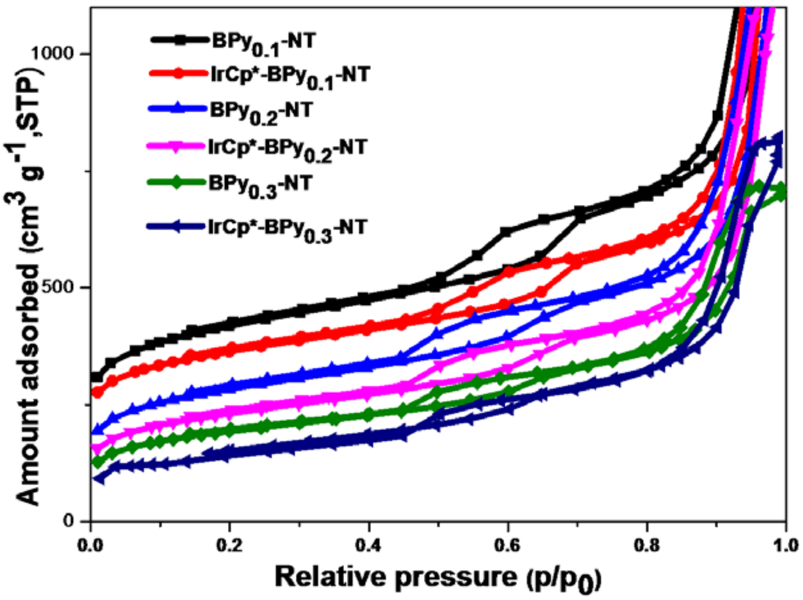

Figure S5. Nitrogen adsorption-desorption isotherms of BPy_x-NT and IrCp*-BPy_x-NT.

The nitrogen adsorption-desorption isotherms of the BPy_x-NT and IrCp*-BPy_x-NT were type IV with a hysteresis loops at relative pressures P/P_0 = 0.5-0.7, which is typical for mesoporous materials (Figure S5). The textural properties of these samples are summarized in Table S2. With the increase of the proportion of bipyridine- to benzene-bridged precursor, the BET surface areas were found to be remarkably decreased from 830 to 455 m^2/g. Meanwhile, the pore diameter was almost maintained as 6 nm. After the formation of Ir complex on the nanotubes, both of the BET surface areas and the pore diameters were slightly decreased, as shown in Figure S6 and Table S2.

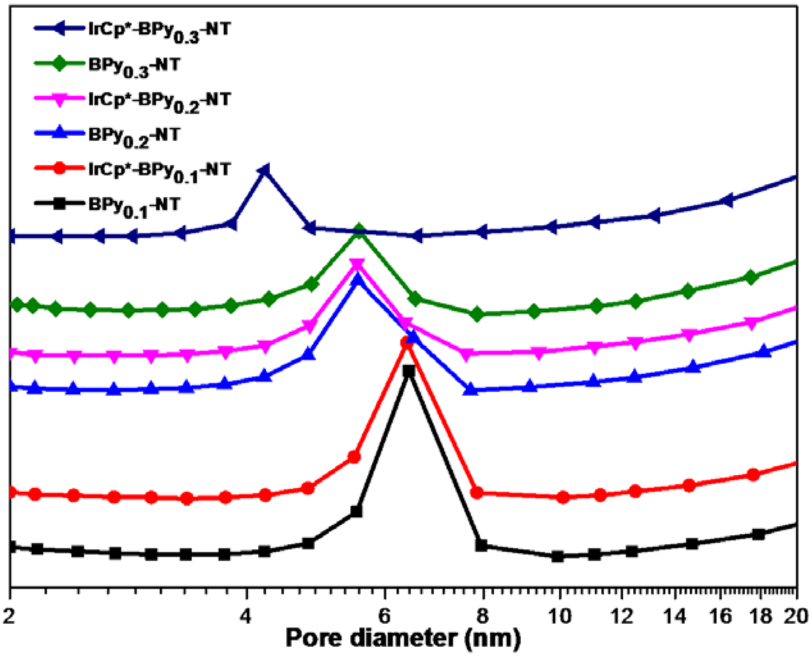

Figure S6. Pore size distributions of BPy_x-NT and IrCp*-BPy_x-NT.

Table S2. Physicochemical properties of different materials

Materials	Surface area[a] (m^2/g)	Pore diameter[b] (nm)
$BPy_{0.1}$-NT	830	6.5
$BPy_{0.2}$-NT	642	5.6
$BPy_{0.3}$-NT	455	5.5
IrCp*-$BPy_{0.1}$-NT	608	6.3
IrCp*-$BPy_{0.2}$-NT	522	5.5
IrCp*-$BPy_{0.3}$-NT	387	4.6

[a]The BET surface areas were calculated from the data in the relative pressure range of P/P_0 = 0.05~0.25; [b]Pore size distributions were calculated from adsorption branch using the BJH method.

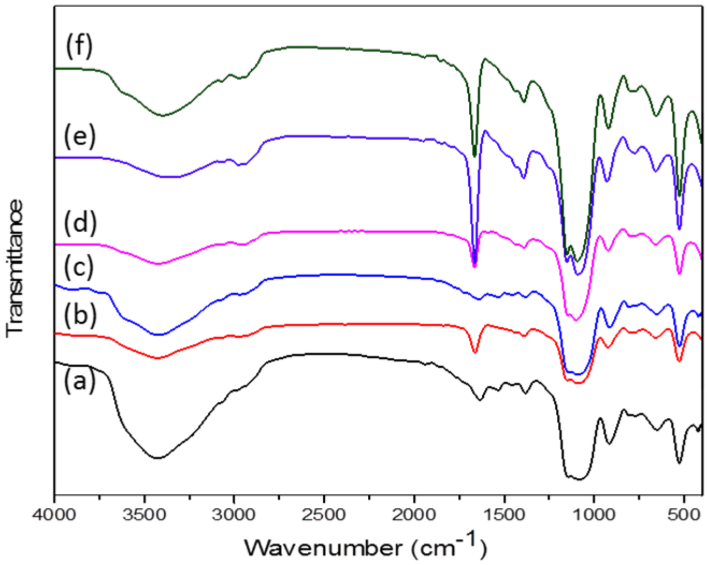

Figure S7. FT-IR spectra of (a) $BPy_{0.1}$-NT, (b) IrCp*-$BPy_{0.1}$-NT, (c) $BPy_{0.2}$-NT, (d) IrCp*-$BPy_{0.2}$-NT, (e) $BPy_{0.3}$-NT, (f) IrCp*-$BPy_{0.3}$-NT.

Figure S7 displays FT-IR spectra of the BPy-NT and IrCp*-BPy-NT with different molar ratio of bipyridine precursors to benzene. For all materials, the band at 1620 cm^{-1} and 3035 cm^{-1} are attributed to the benzene ring C=C vibration and the stretching modes of C-H species of aromatic moiety, respectively. The stretching and bending vibration of the surface silanol groups appeared at 3440 and 1630 cm^{-1}, respectively. The bands at 1150 cm^{-1} was assigned to the Si-C vibrations and a band at 2800 cm^{-1} was attributed to the asymmetric and symmetric stretching vibration of $-CH_2-$ bonds. In addition, the weak bands at 1480 and 780 cm^{-1} represent $-CH_2-$ bending and $-CH_2-$ rocking modes, respectively.

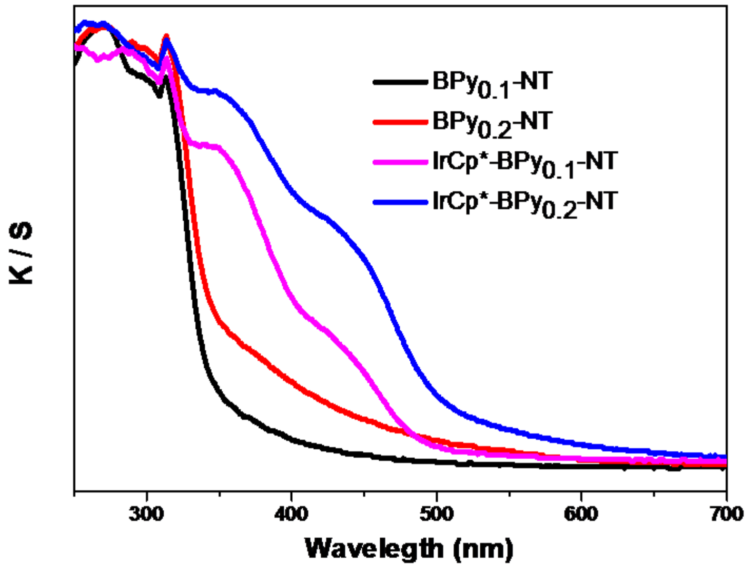

Figure S8. UV/vis diffuse reflectance spectra of $BPy_{0.1}$-NT, $BPy_{0.2}$-NT, IrCp*-$BPy_{0.1}$-NT and IrCp*-$BPy_{0.2}$-NT.

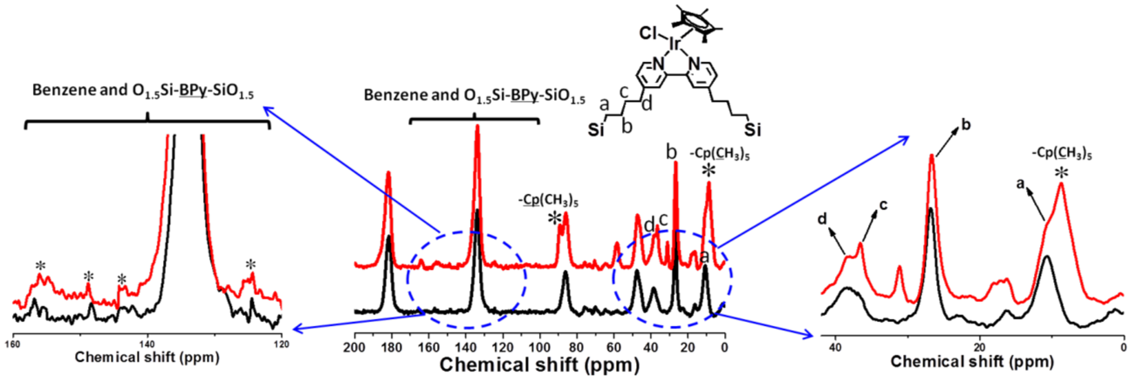

Figure S9. ^{13}C CP MAS NMR spectra of $BPy_{0.3}$-NT (black line) and IrCp*-$BPy_{0.3}$-NT (red line).

In Figure S9, very small peaks at 120-160 ppm are assigned to the bipyridine groups, in addition to a strong peak at 133 ppm due to the phenyl groups. 9 and 90 ppm (* marked, middle figure) belong to two kinds of C in Cp*. The peaks of C (*a, b, c, d*) in the linker can be found at 10.8, 26.5, 36.3, 38.6 ppm, respectively.

5. Synthesis of IrCp*-Gbpy-NT

Organosilica nanotubes (NT) without bipyridine incorporation in the framework were synthesized according to our previous report.[3a] 0.55 g of P123 and 1.75 g of KCl were dissolved in different volume of 2 M HCl at 38 °C. After the copolymer was fully dissolved, 3.50 mmol of BTEB was added with vigorous stirring for 6 min. And then the resultant mixture was stirred for additional 24 h at the same temperature. After that, the mixture was aged at 100 °C under static conditions for an additional 24 h. The solid product was recovered by filtration and air-dried at room temperature overnight. Finally, the surfactant was extracted by refluxing 1.0 g of as-synthesized material in 200 mL of ethanol containing 1.27 g of HCl solution for 24 h. The surfactant-free samples were denoted as BNTs. The synthetic process of bipyridine-grafted BNT was as follows: 0.35 g BNTs and 0.195 mmol bipyridine-based precursor **(1)** were added in 100 mL three-neck flask under nitrogen, and then 30 mL dehydrated toluene was added. After the reaction mixture was refluxed for 24 h, the suspension was filtered and washed with toluene and ethanol. For the synthesis of NTs grafted Ir-complex, 100 mg bipyridine-grafted BNTs and 10 mg $[Cp^*IrCl(\mu\text{-}Cl)]_2$ were added to 60 mL anhydrous ethanol under nitrogen. After the suspension was stirred under refluxing conditions for 24 h, the product was obtained by filtration and washed with DMF and distilled water to remove unreacted $[Cp^*IrCl(\mu\text{-}Cl)]_2$. The final sample was denoted as IrCp*-Gbpy-NT.

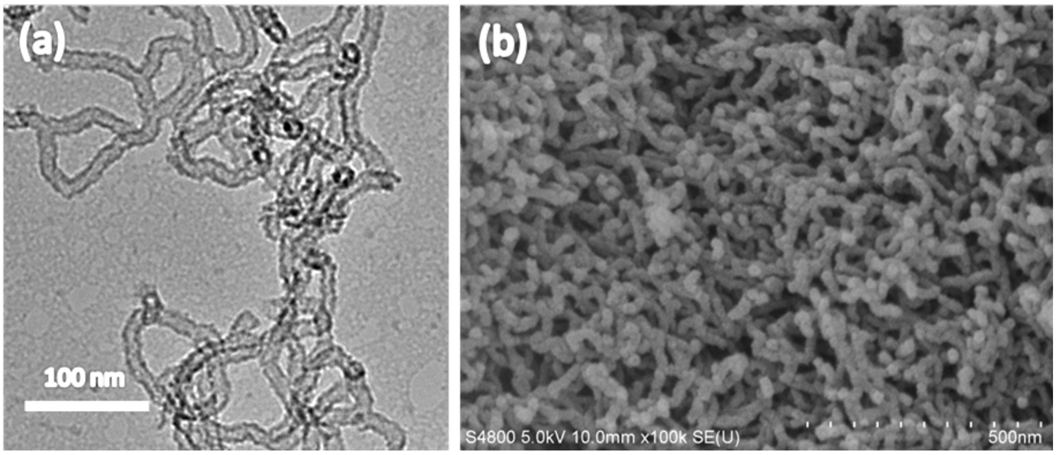

Figure S10. (a) TEM and (b) SEM images of IrCp*-Gbpy-NT.

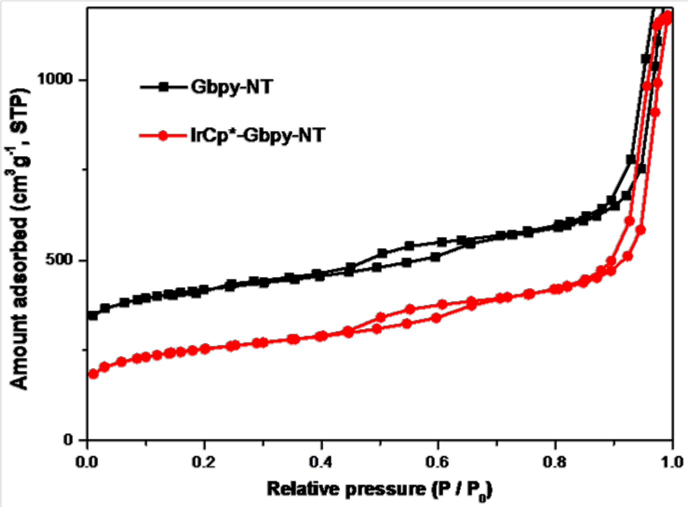

Figure S11. Nitrogen adsorption-desorption isotherms of Gbpy-NT and IrCp*-Gbpy-NT.

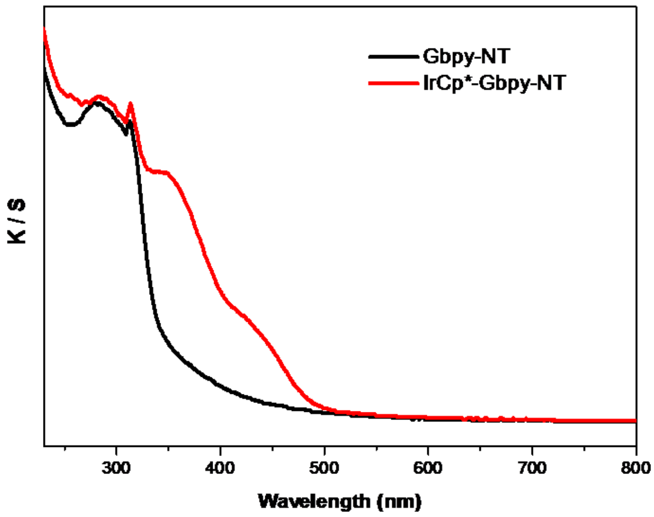

Figure S12. UV/vis diffuse reflectance spectra of Gbpy-NT and IrCp*-Gbpy-NT.

The new peaks of IrCp*-Gbpy-NT at at around λ = 360 and 440 nm indicate the successful grafting of $[IrCp^*Cl(bpy)]^+$ on the BNTs.

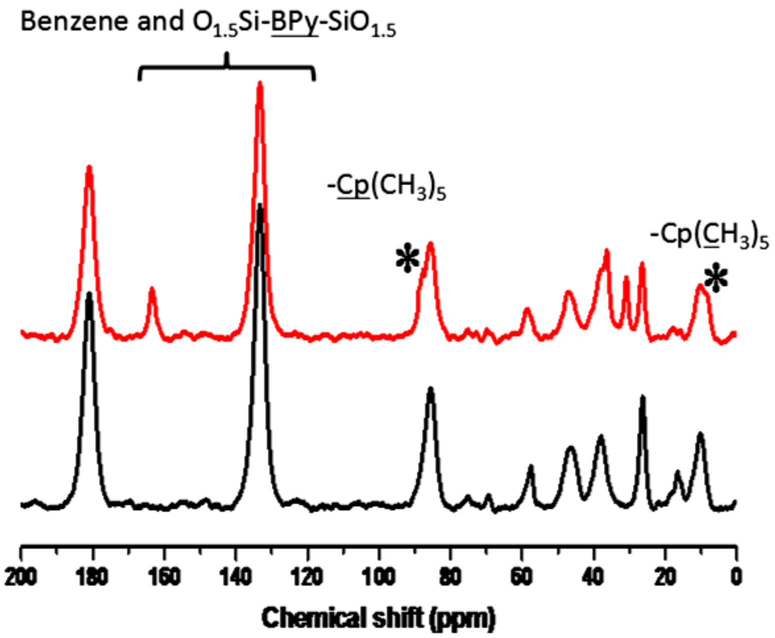

Figure S13. ^{13}C CP MAS NMR spectra of Gbpy-NT (black line) and IrCp*-Gbpy-NT (red line).

The new signals of IrCp*-Gbpy-NT at 9 and 90 ppm (star-marked) are assigned to Cp* ligand in $[IrCp^*Cl(bpy)]^+$ complex, compared to that of Gbpy-NT.

6. Synthesis of IrCp*-BPy-SBA-15

BPy-SBA-15 was synthesized according to the previous report with some minor modification.[4] In a typical synthesis, 0.97 g of P123 was dissolved in 35 mL H_2O and 0.22 mL HCl at 40 °C. After stirring for 2 h, 2.487 mmol of BTEB was added with vigorous stirring. After stirring for 2 h at 40 °C, 0.2764 mmol of **(1)** was added dropwise. And then the resultant mixture was stirred for additional 2 h at the same temperature. After that, the mixture was aged at 100 °C under static conditions for an additional 24 h. The solid product was recovered by filtration and air-dried at room temperature overnight. Finally, the surfactant was extracted by refluxing 1.0 g of as-synthesized material in 200 mL of ethanol containing 4 mL of HCl solution for 24 h. The surfactant-free samples were denoted as BPy-SBA-15. For the synthesis of Ir_1-BPy-SBA-15, 100 mg BPy-SBA-15 and 10 mg $[Cp^*IrCl(\mu\text{-}Cl)]_2$ were added to 60 mL anhydrous ethanol under nitrogen. After the suspension was stirred under refluxing conditions for 24 h, the product was removed by filtration and washed with DMF and distilled water to remove unreacted $[Cp^*IrCl(\mu\text{-}Cl)]_2$. The final sample was denoted as IrCp*-BPy-SBA-15.

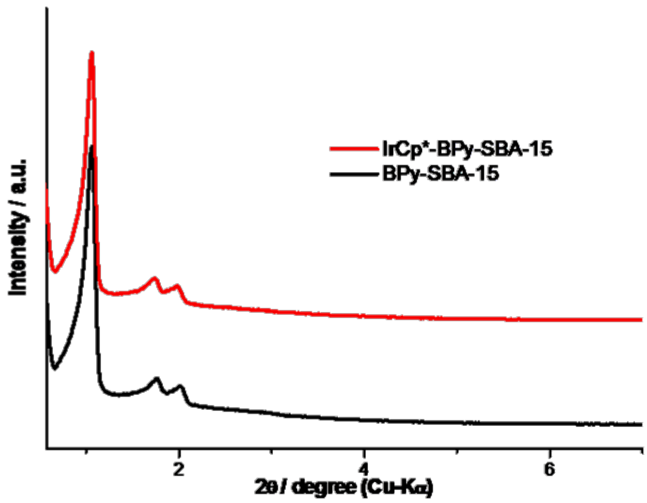

Figure S14. XRD patterns of BPy-SBA-15 and IrCp*-BPy-SBA-15.

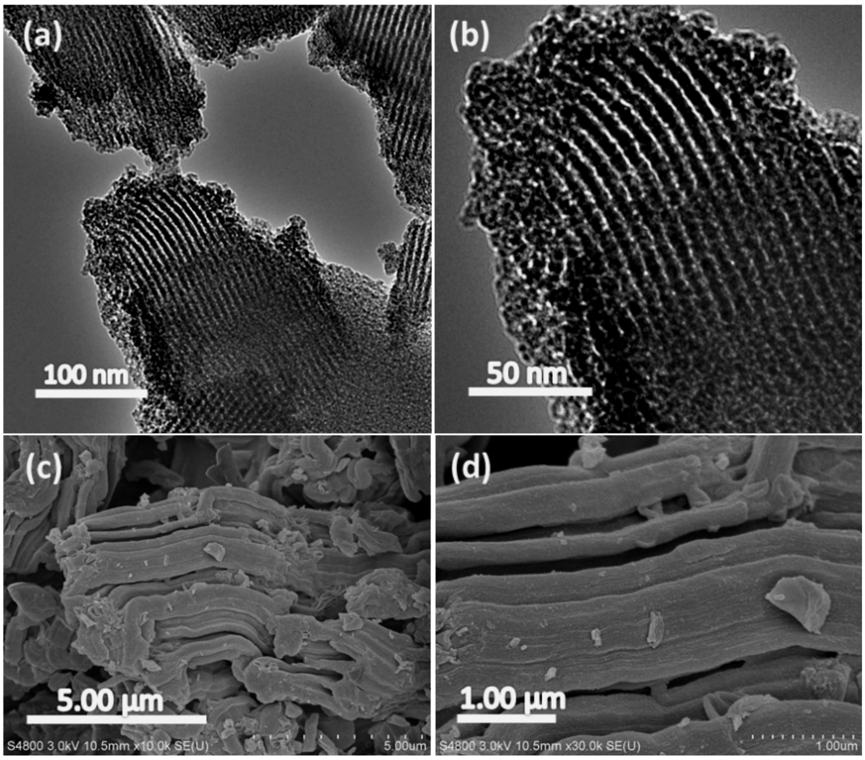

Figure S15. (a), (b) TEM and (c), (d) SEM images of IrCp*-BPy-SBA-15.

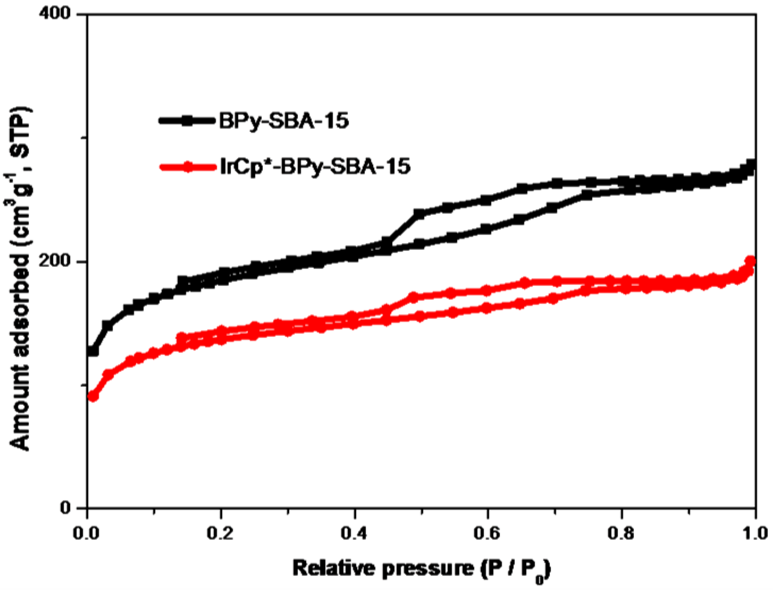

Figure S16. Nitrogen adsorption-desorption isotherms of BPy-SBA-15 and IrCp*-BPy-SBA-15.

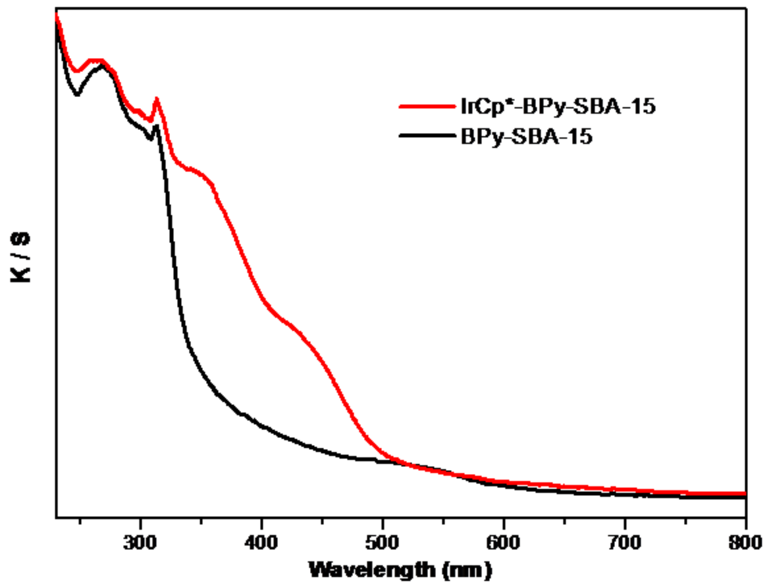

Figure S17. UV/vis diffuse reflectance spectra of BPy-SBA-15 and IrCp*-BPy-SBA-15.

The new peaks of IrCp*-BPy-SBA-15 at at around λ = 360 and 440 nm indicate the successful formation of $[IrCp*Cl(bpy)]^+$ on the support.

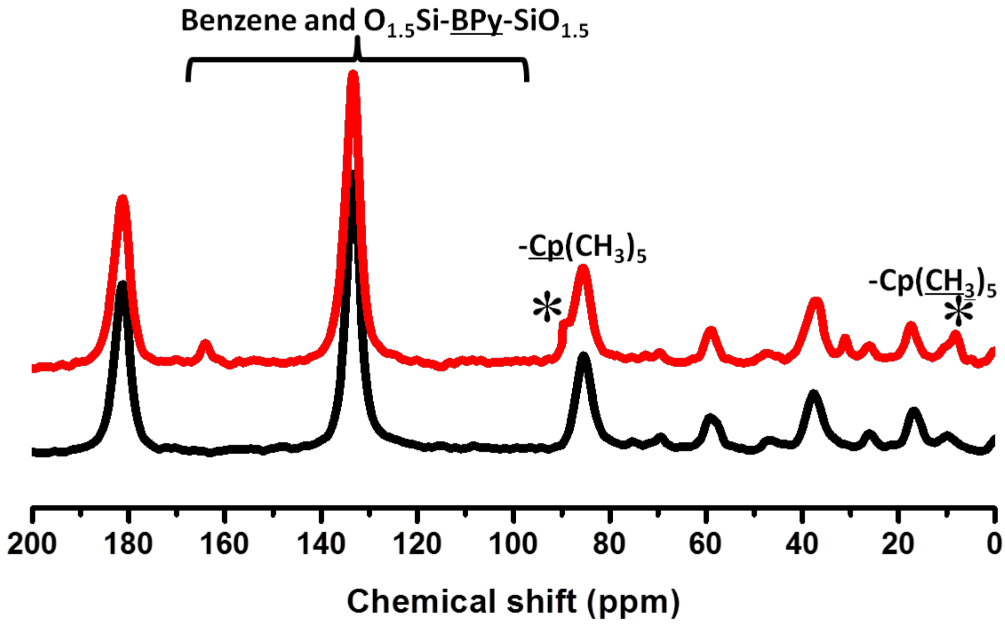

Figure S18. ^{13}C CP MAS NMR spectra of BPy-SBA-15 and IrCp*-BPy-SBA-15.

The new signals of IrCp*-BPy-SBA-15 at 9 and 90 ppm (star-marked) are assigned to Cp* ligand in $[IrCp^*Cl(bpy)]^+$ complex, compared to that of BPy-SBA-15.

7. Synthesis of Ir(cod)-BPy$_{0.3}$-NT

The iridium-immobilized nanotube catalysts were prepared by adding BPy$_{0.3}$-NT (100 mg) to a solution of $[Ir(OMe)(cod)]_2$ (5 mg) in 20 ml dry benzene under an nitrogen atmosphere. After the suspension was stirred under room temperature conditions for 12 h, the solid phase was obtained by filtration and washed with dry benzene to remove unreacted $[Ir(OMe)(cod)]_2$. The dried samples were named as Ir(cod)-BPy$_{0.3}$-NT. The Ir amount was 0.135 mmol/g for Ir(cod)-BPy$_{0.3}$-NT from Energy-dispersive X-ray spectroscopy (EDX) analysis.

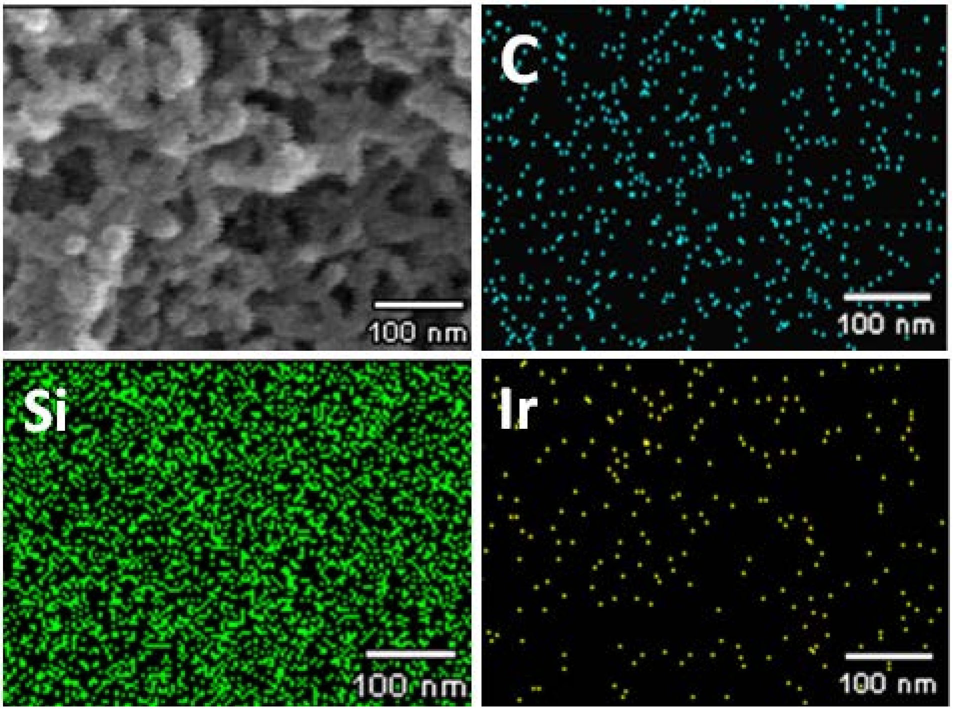

Figure S19. EDX analysis of Ir(cod)-BPy$_{0.3}$-NT.

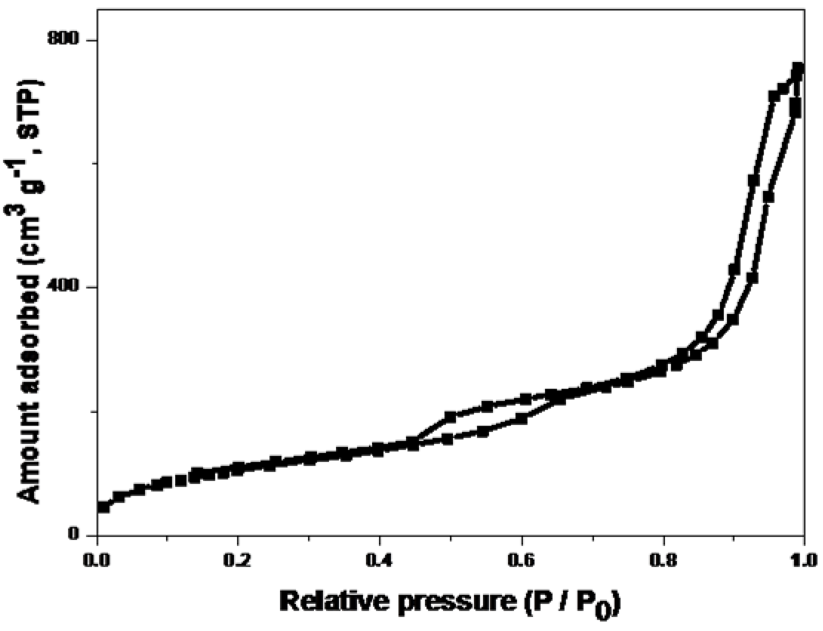

Figure S20. Nitrogen adsorption/desorption isotherm of Ir(cod)-BPy$_{0.3}$-NT.

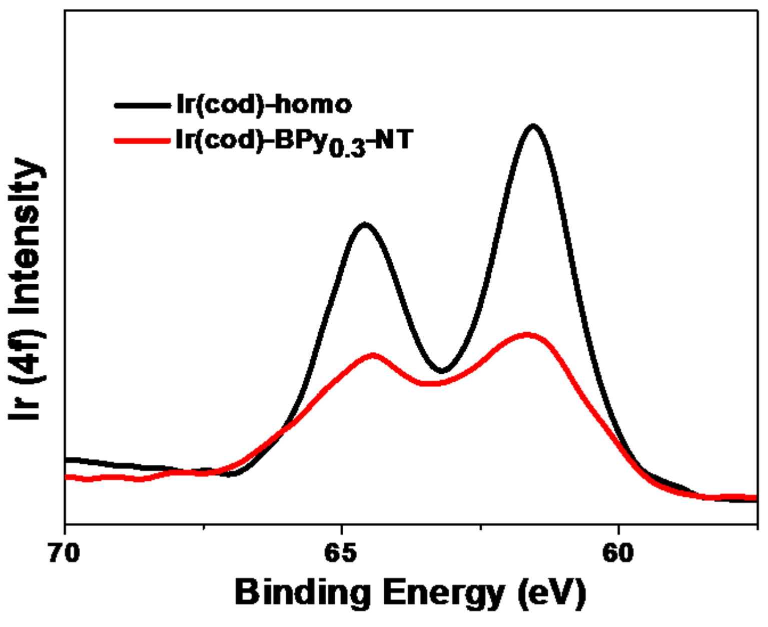

Figure S21. The Ir(4f) XPS analysis of homogeneous catalyst Ir(cod)-homo and

Ir(cod)-BPy$_{0.3}$-NT.

8. Synthesis of Ir(cod)-Gbpy-NT

For the synthesis of nanotube-based grafting Ir-complex, 100 mg bipyridine-grafted BNTs and 5 mg $[Ir(OMe)(cod)]_2$ were added to 20 mL dry benzene under nitrogen. After the suspension was stirred under room temperature conditions for 12 h, the product was removed by filtration and washed with dry benzene to remove unreacted $[Ir(OMe)(cod)]_2$. The final sample was denoted as Ir(cod)-Gbpy-NT.

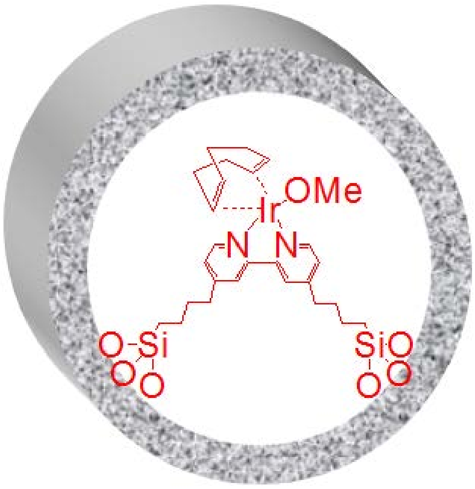

Scheme S1. Schematic representation of Ir(cod)-Gbpy-NT.

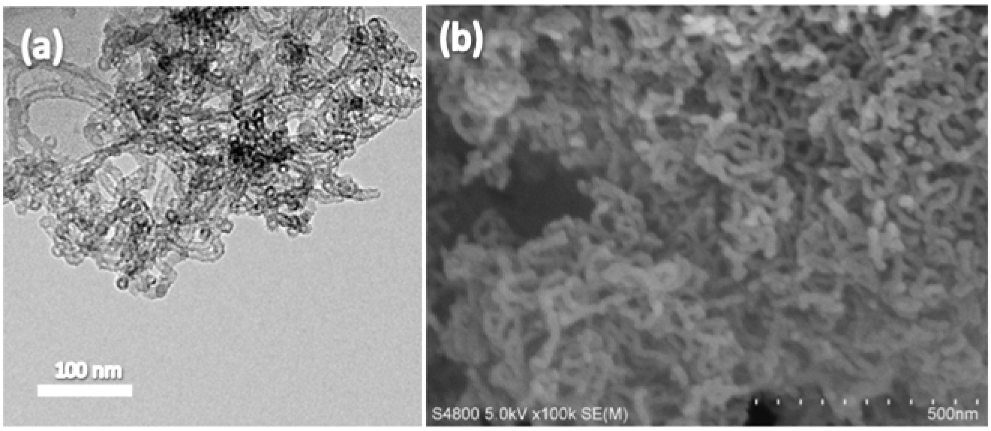

Figure S22. (a) TEM and (b) SEM images of Ir(cod)-Gbpy-NT.

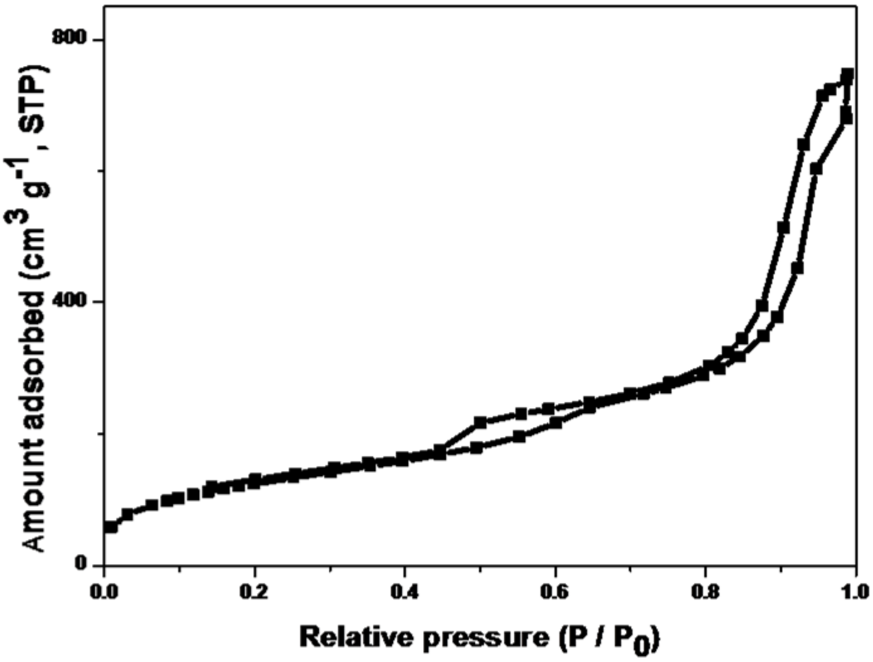

Figure S23. Nitrogen adsorption-desorption isotherm of Ir(cod)-Gbpy-NT.

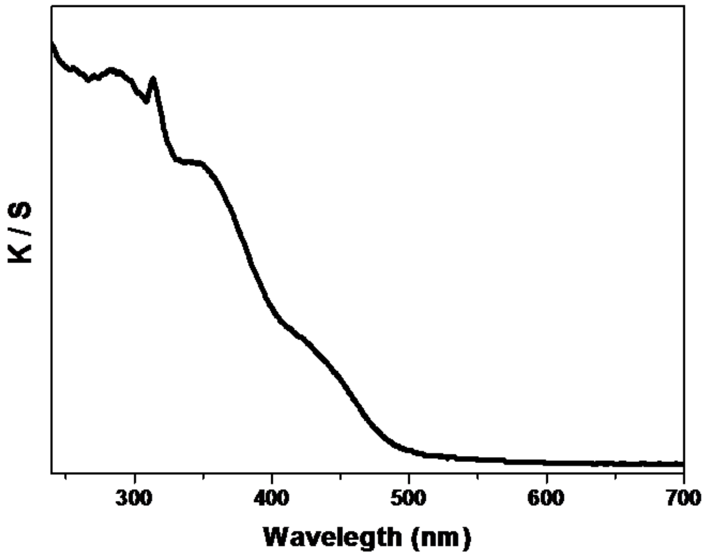

Figure S24. UV/vis diffuse reflectance spectrum of Ir(cod)-Gbpy-NT.

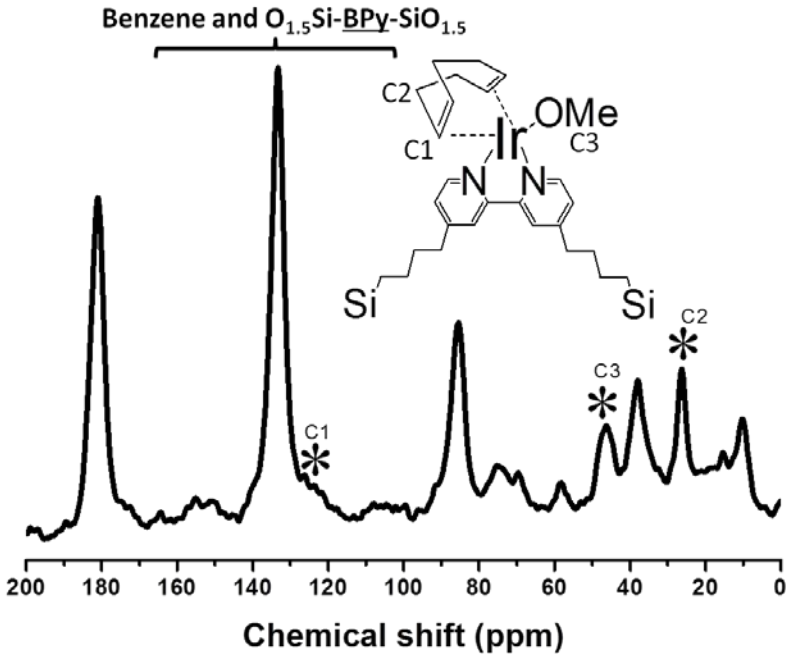

Figure S25. ^{13}C CP MAS NMR spectrum of Ir(cod)-Gbpy-NT.

9. Synthesis of Ir(cod)-BPy-SBA-15

For the synthesis of Ir(cod)-BPy-SBA-15, 100 mg BPy-SBA-15 and 5 mg $[Ir(OMe)(cod)]_2$ were added to 20 mL dry benzene under nitrogen. After the suspension was stirred under room temperature conditions for 12 h, the product was removed by filtration and washed with dry benzene to remove unreacted $[Ir(OMe)(cod)]_2$. The final sample was denoted as Ir(cod)-BPy-SBA-15.

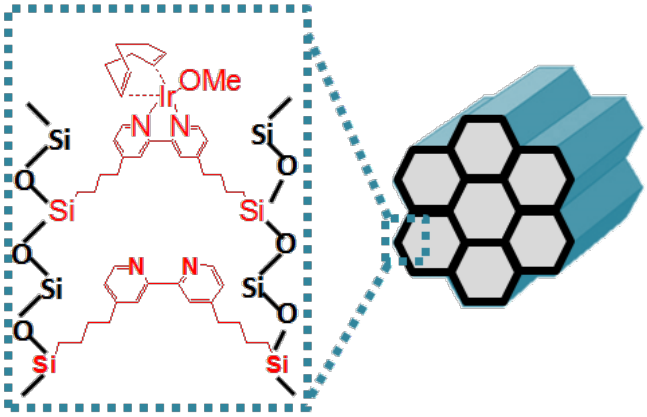

Scheme S2. Schematic representations of Ir(cod)-BPy-SBA-15.

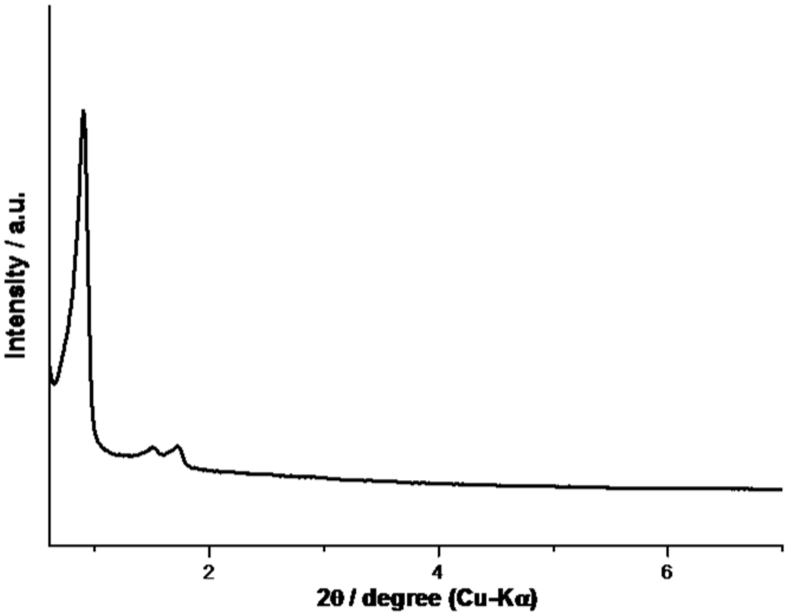

Figure S26. XRD pattern of Ir(cod)-BPy-SBA-15.

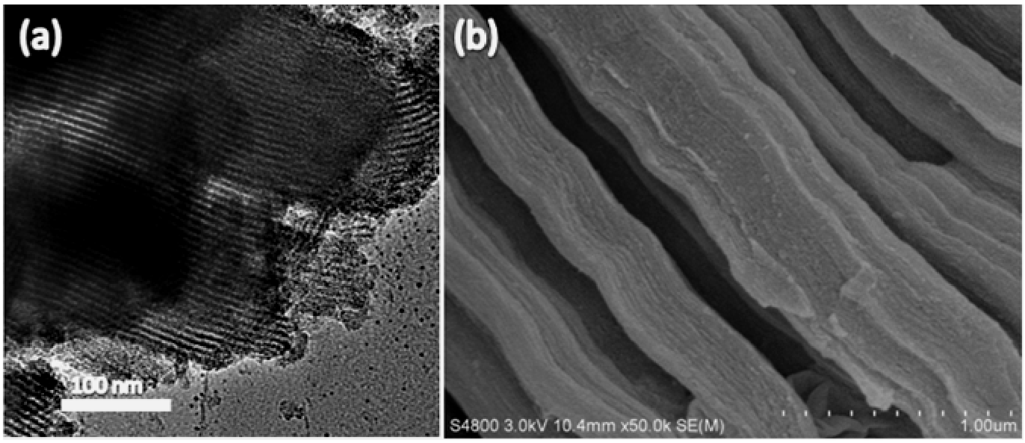

Figure S27. (a) TEM and (b) SEM images of Ir(cod)-BPy-SBA-15.

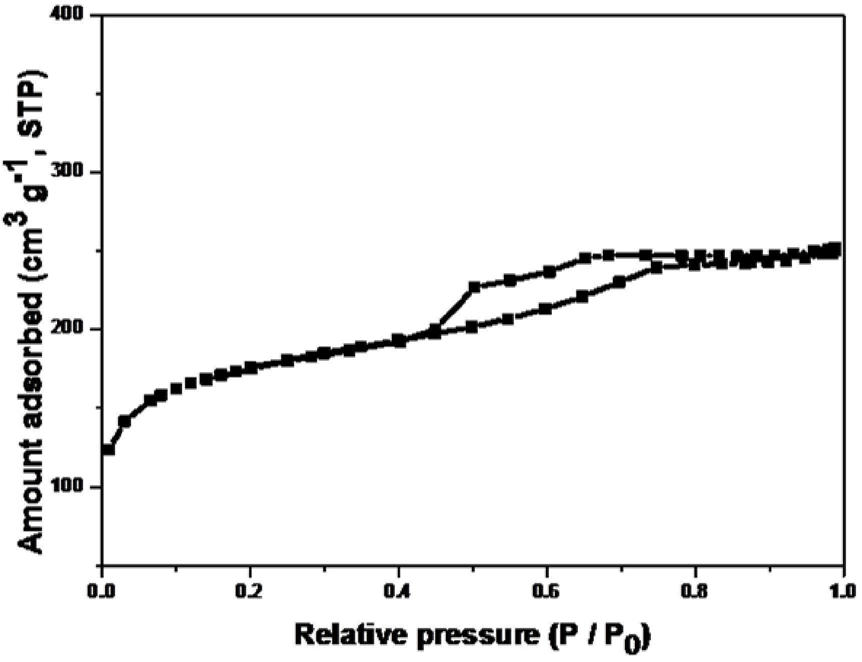

Figure S28. Nitrogen adsorption/desorption isotherm of Ir(cod)-BPy-SBA-15.

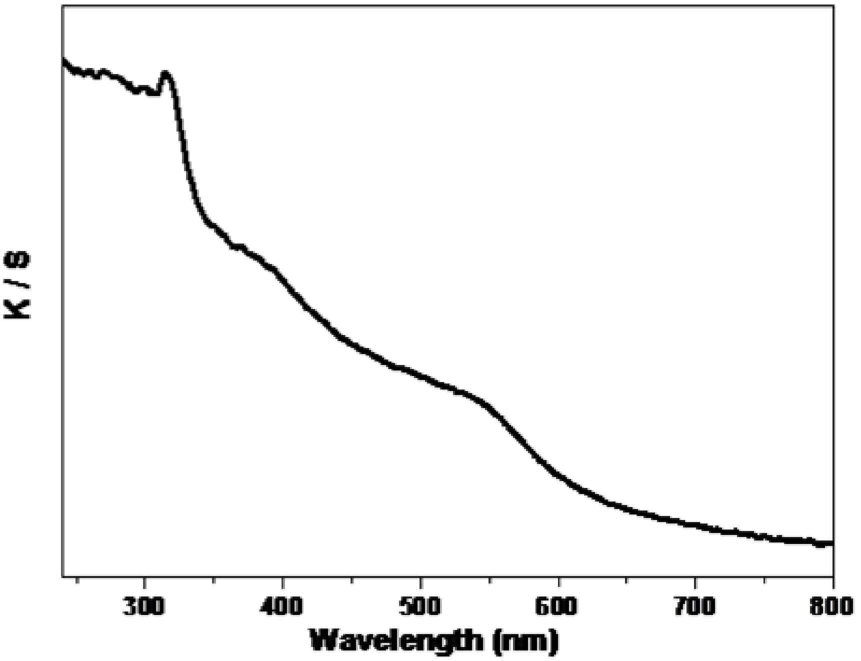

Figure S29. UV/vis diffuse reflectance spectrum of Ir(cod)-BPy-SBA-15.

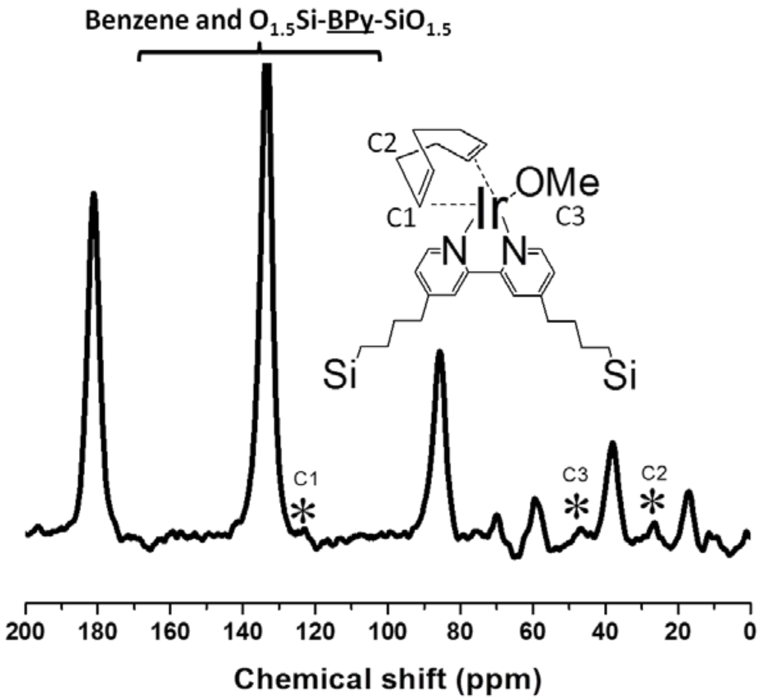

Figure S30. ^{13}C CP MAS NMR spectrum of Ir(cod)-BPy-SBA-15.

Table S3. Physicochemical properties of different materials.

Materials	Surface area[a] (m^2/g)	Pore diameter[b] (nm)
BPy-SBA-15	540	4.6
Gbpy-NT	780	6.3
IrCp*-BPy-SBA-15	465	4.2
IrCp*-Gbpy-NT	600	5.6
Ir(cod)-$BPy_{0.3}$-NT	369	4.5
Ir(cod)-BPy-SBA-15	440	4.0
Ir(cod)-Gbpy-NT	587	5.4

[a]The BET surface areas were calculated using the data in the relative pressure range of P/P_0 = 0.05~0.25; [b]Pore size distributions were calculated from adsorption branch using the BJH method.

10. Ir-BPy-NT-catalyzed C-H activation reactions

C-H oxidation reaction of THF:

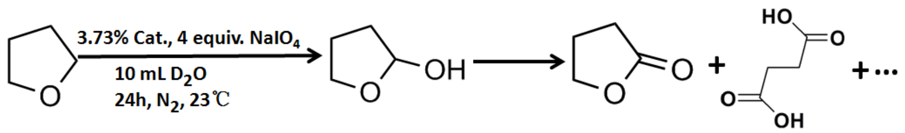

In a typical experiment, 25 mg IrCp*-BPy$_{0.3}$-NT (3.73% catalyst loading) was added to a solution containing 45 µL THF (0.6 mmol, limiting reagent) and 0.5136 g $NaIO_4$ oxidant (4 equiv, 2.4 mmol) in 10 mL D_2O with sodium d$_4$-trimethylsilyl propanoate (3 mg) as internal standard under N_2 at room temperature. A sample (~ 1 mL) of the reaction solution was collected for direct ^{1}H NMR analysis. Products were identified by comparing with ^{1}H NMR spectra of authentic samples. The error is about 5%.

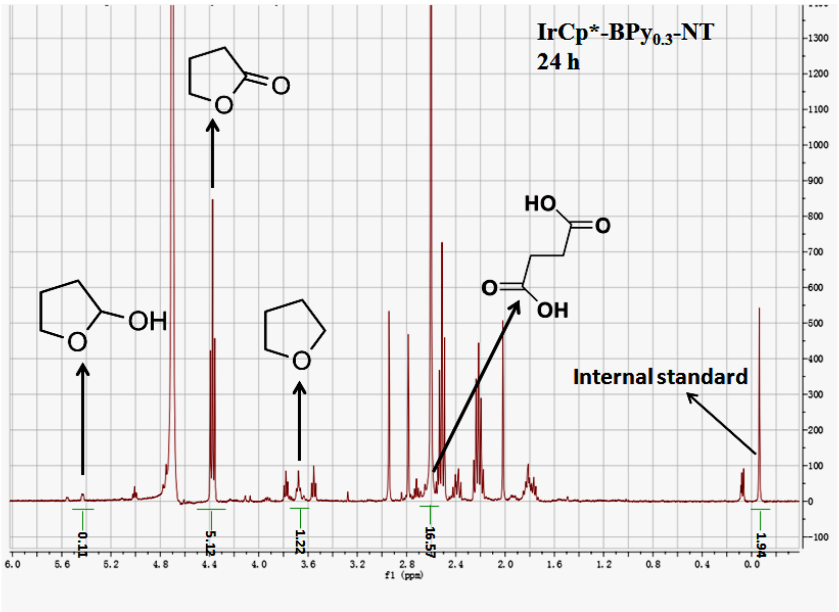

Figure S31. ^{1}H NMR analysis of THF oxidation for IrCp*-BPy$_{0.3}$-NT at 24 h.

The C-H oxidation reactions of other substrates (cyclohexane, cyclooctene, ethylbenzene, pyrrolidine) also were tested. General procedure, 25 mg IrCp*-BPy$_{0.3}$-NT (3.73% catalyst loading) was added to a solution containing 0.6 mmol substrates and 0.5136 g $NaIO_4$ oxidant in 10 mL 1:1 $^tBuOH/D_2O$ with sodium d_4-trimethylsilyl propanoate (3 mg) as internal standard under N_2 at room temperature. A sample (~ 1 mL) of the reaction solution was collected for direct ^1H NMR analysis. Products were identified by comparing with ^1H NMR spectra of authentic samples.

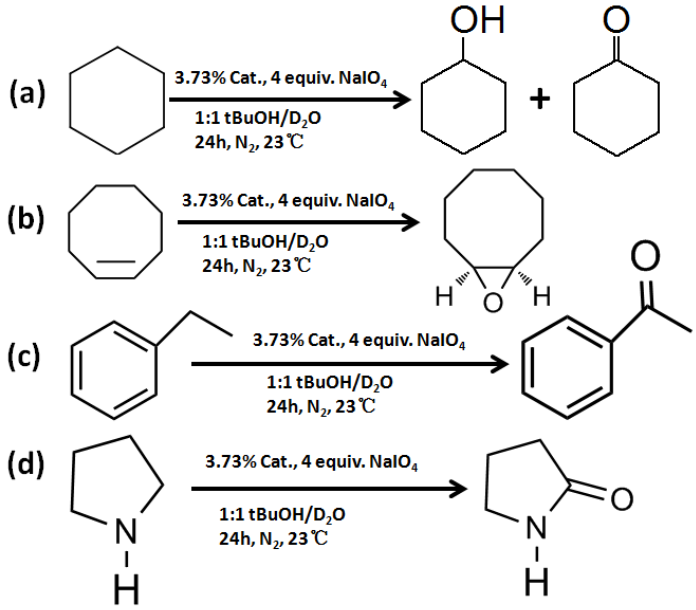

Figure S32. The C-H oxidation reaction of different substrates.

C-H borylation reaction:

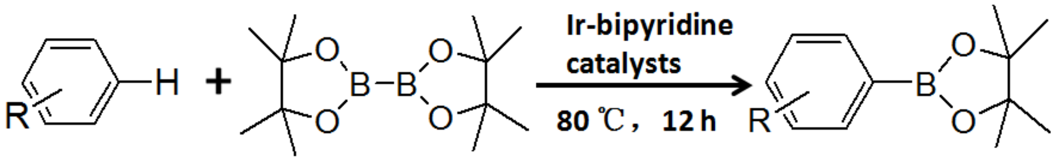

Figure S33. The C-H borylation reaction of arenes.

In a typical experiment, Ir(cod)-$BPy_{0.3}$-NT (0.005 mmol Ir) and bis(pinacolato)diboron (B_2pin_2) (0.33 mmol) was added to arene (20 mmol) under N_2. Then the mixture was stirred at 80 °C. At suitable time intervals, part of solution were removed by syringe and filtered by membrane filter (0.22 μm) and diluted with chloroform-*d* containing internal standard ($SiMe_4$). The yield of pinacol phenylboronate was analyzed by ^{1}H NMR spectroscopy. Products were identified by comparing with ^{1}H NMR spectra of authentic samples.

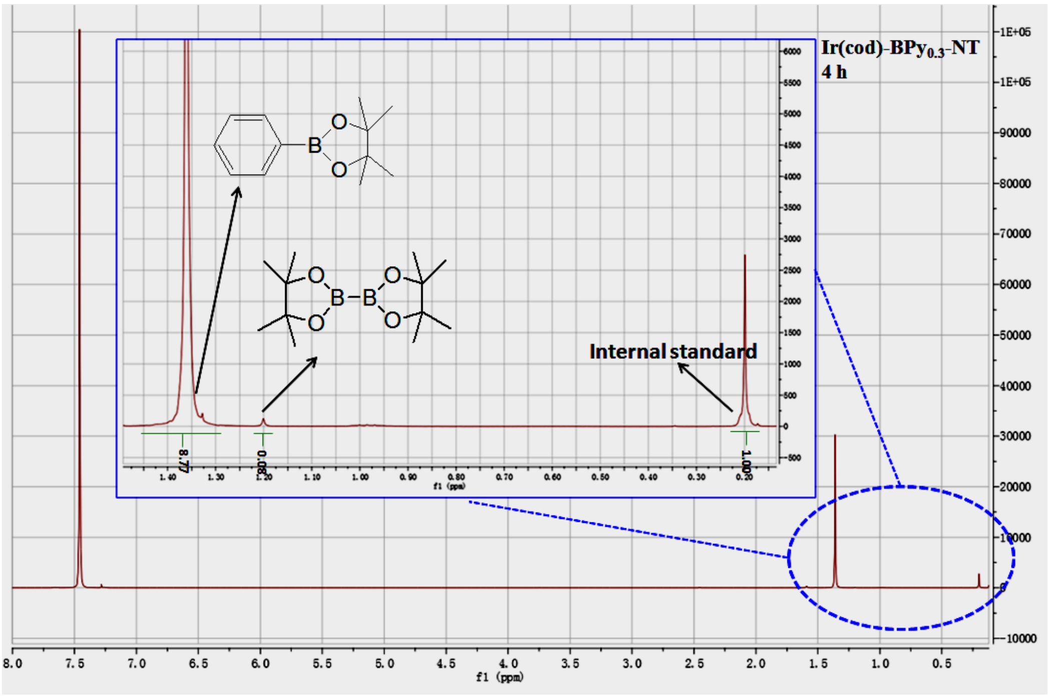

Figure S34. ^{1}H NMR analysis of benzene borylation for Ir(cod)-$BPy_{0.3}$-NT at 4 h.

11. Reusability test for C-H activation reaction

C-H oxidation reaction:

In a typical experiment, 25 mg fresh IrCp*-$BPy_{0.3}$-NT catalyst was added to a solution containing 45 μL THF and 0.5136 g $NaIO_4$ oxidant in 10 mL D_2O with sodium d_4-trimethylsilyl propanoate (3 mg) as internal standard under N_2 at room temperature. After every 24 h, the catalyst was recovered by simple filtration and was used for the next reaction. For comparison, the recycling catalytic performance was also carried out for the homogeneous catalyst. After every 24 h, a certain amount of THF and $NaIO_4$ oxidant were added to the reaction system to make it back to the initial concentration. Finally, the heterogeneous catalyst was recovered by filtration to measure their structural properties after four cycles and the homogeneous catalyst was also characterized to detect the results of oxidation.

C-H borylation reaction:

In a typical experiment, Ir(cod)-BPy$_{0.3}$-NT (0.005 mmol Ir) and bis(pinacolato)diboron (B_2pin_2) (0.33 mmol) was added to arene (20 mmol) under N_2. Then the mixture was stirred at 80 °C. After every 4 h, the catalyst was recovered by simple filtration and used for the next reaction.

12. The analysis of homogeneous catalyst by using mass spectrum

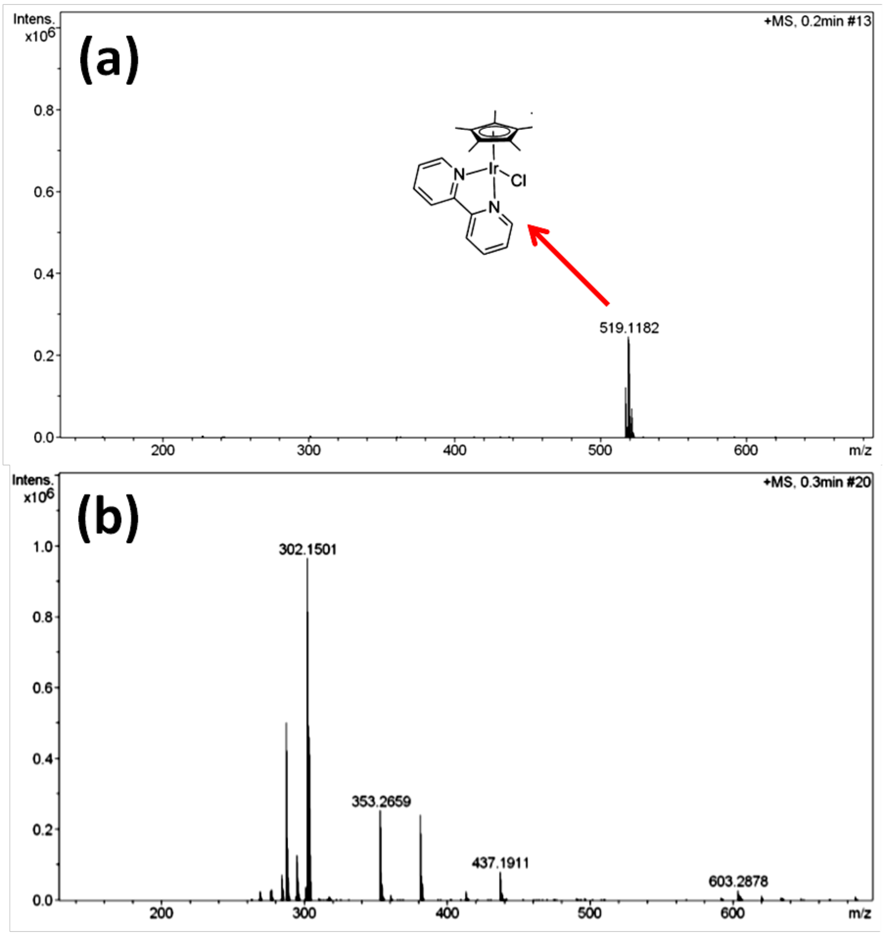

Figure S35. The mass spectrum (MS) analysis of before (a) and after (b) of C-H oxidation of IrCp*-homo catalyst.

13. The structure analysis of Ir-BPy$_{0.3}$-NT after the reaction

Ir-BPy$_{0.3}$-NT after C-H oxidation reaction:

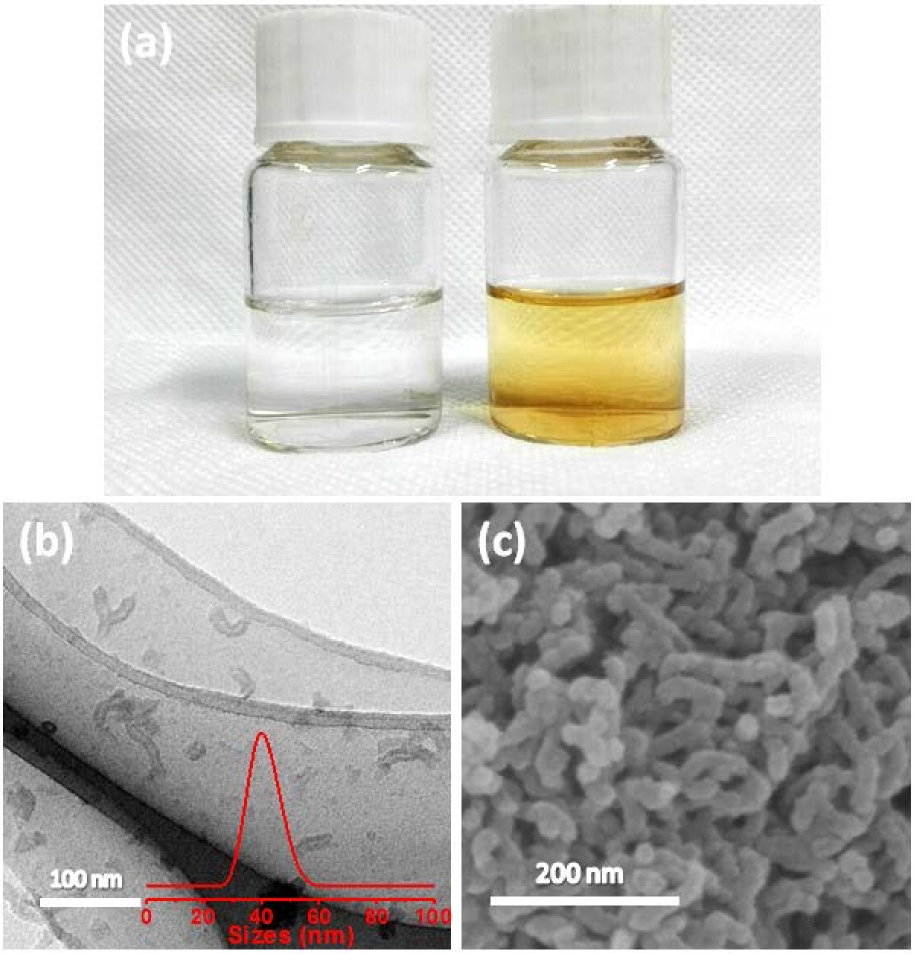

Figure S36. (a) The filtrate of heterogeneous reaction after the fifth reaction (left, colorless) and homogeneous reaction (right, yellow) after the reaction; (b) TEM and (c) SEM images of IrCp*-BPy$_{0.3}$-NT after the fifth reaction.

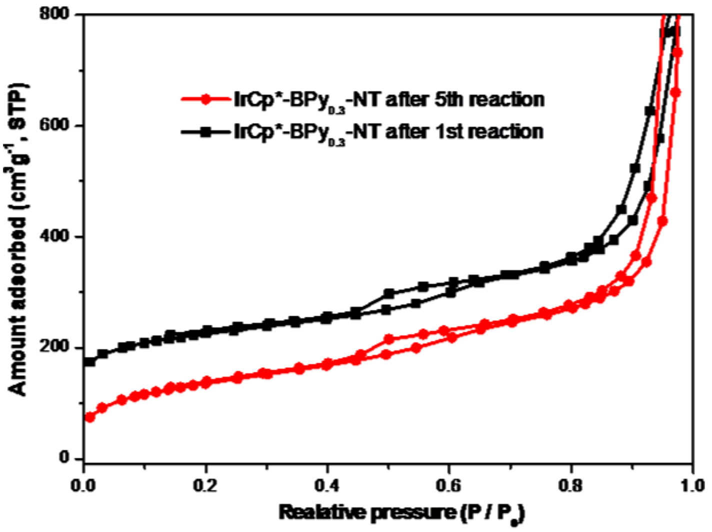

Figure S37. Nitrogen adsorption-desorption isotherms of IrCp*-BPy$_{0.3}$-NT after the first and fifth reactions. (The BET surface areas of IrCp*-BPy$_{0.3}$-NT after the first reaction: 360 m^2 / g, fifth reaction: 342 m^2 / g).

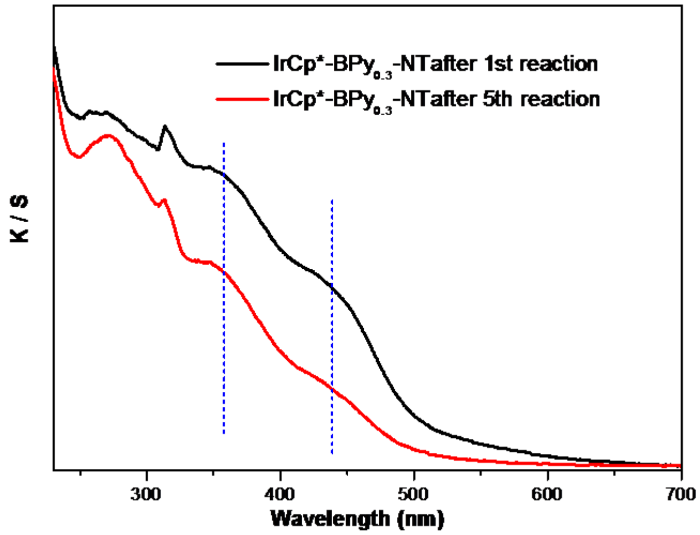

Figure S38. UV/vis diffuse reflectance spectra of IrCp*-BPy$_{0.3}$-NT after the first and fifth reactions.

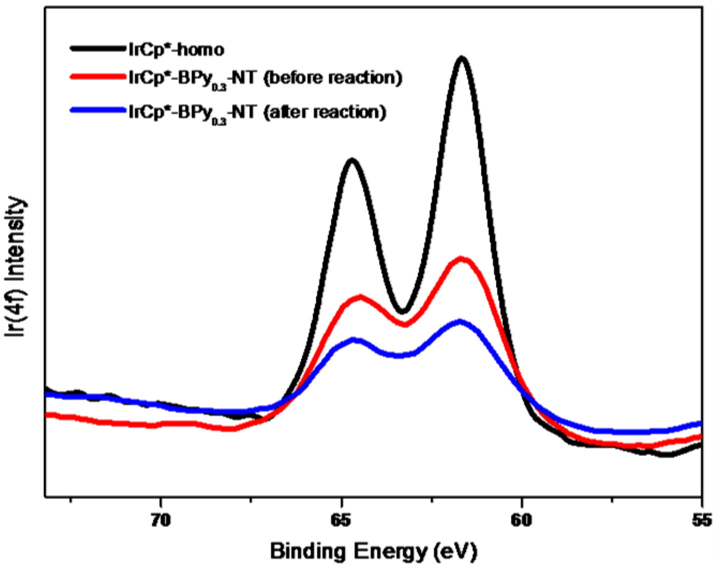

Figure S39. The Ir(4f) XPS analysis of homogeneous catalyst [IrCp*Cl(bpy)]Cl, IrCp*-BPy$_{0.3}$-NT (before reaction) and IrCp*-BPy$_{0.3}$-NT (after the fifth reaction).

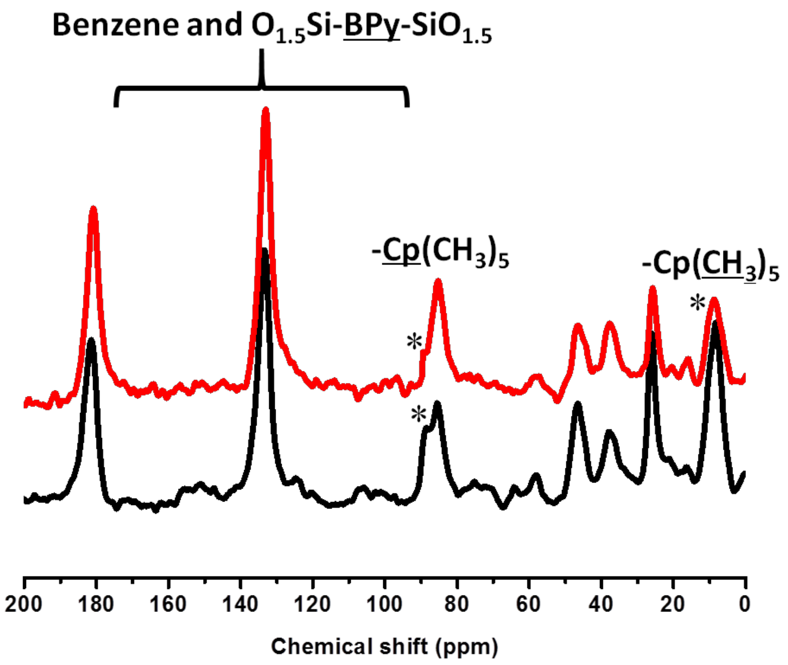

Figure S40. ^{13}C CP MAS NMR spectra of IrCp*-BPy$_{0.3}$-NT after the first (black line) and fifth reactions (red line).

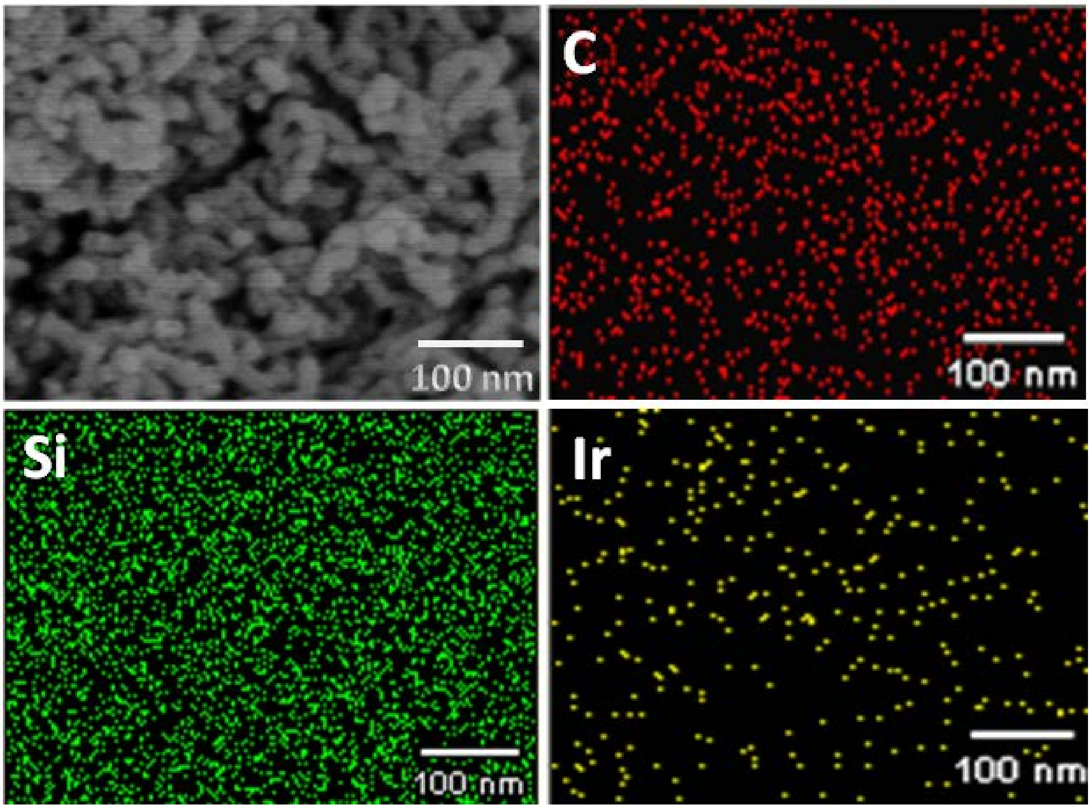

Figure S41. EDX analysis of IrCp*-BPy$_{0.3}$-NT after the fifth reaction.

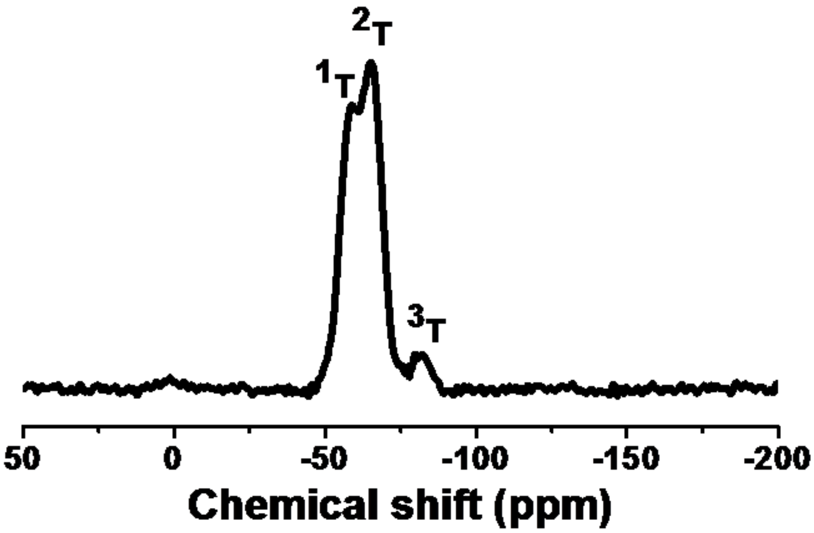

Figure S42. ^{29}Si MAS NMR spectrum of IrCp*-BPy$_{0.3}$-NT after the fifth reaction.

Ir(cod)-BPy$_{0.3}$-NT after C-H borylation reaction:

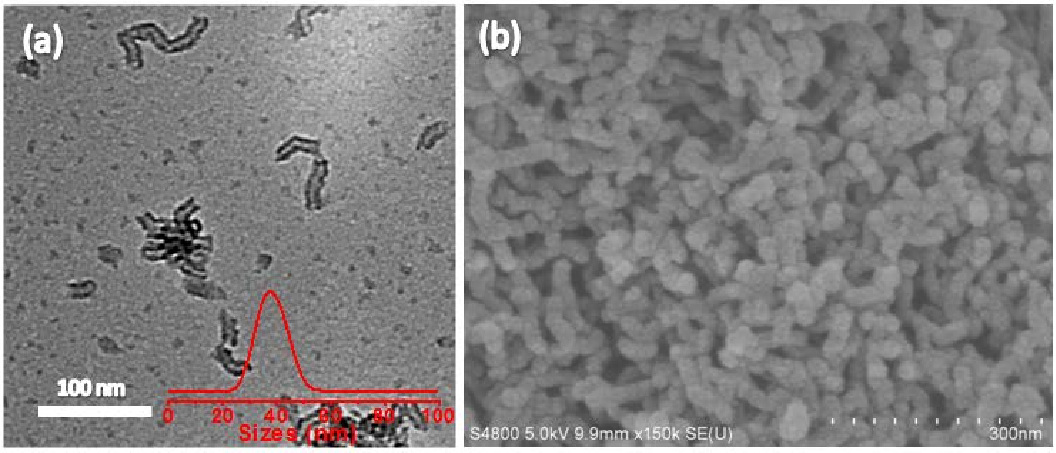

Figure S43. (a) TEM and (b) SEM images of Ir(cod)-BPy$_{0.3}$-NT after the 10th reaction.

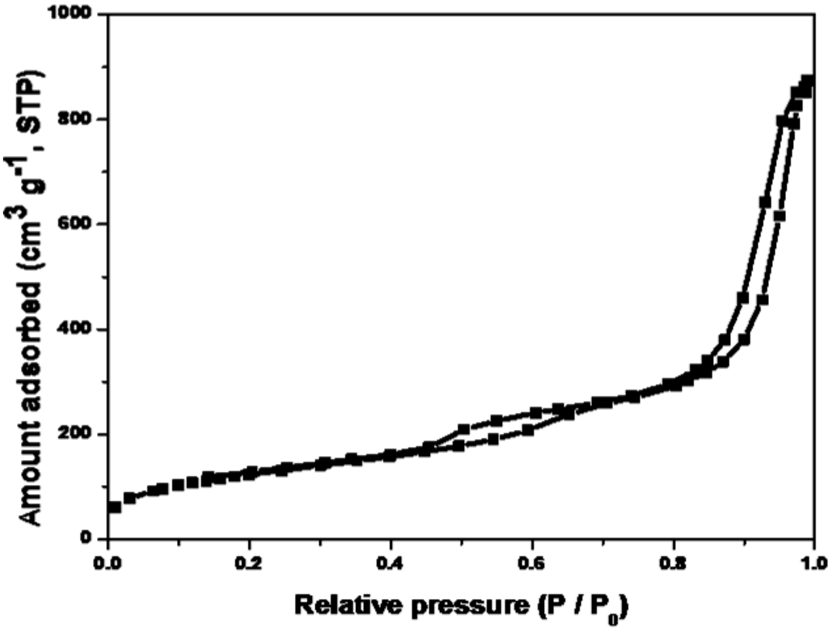

Figure S44. Nitrogen adsorption-desorption isotherm of Ir(cod)-BPy$_{0.3}$-NT after the 10th reaction.

(The BET surface areas of Ir(cod)-BPy$_{0.3}$-NT after the 10th reaction: 328 m^2 / g).

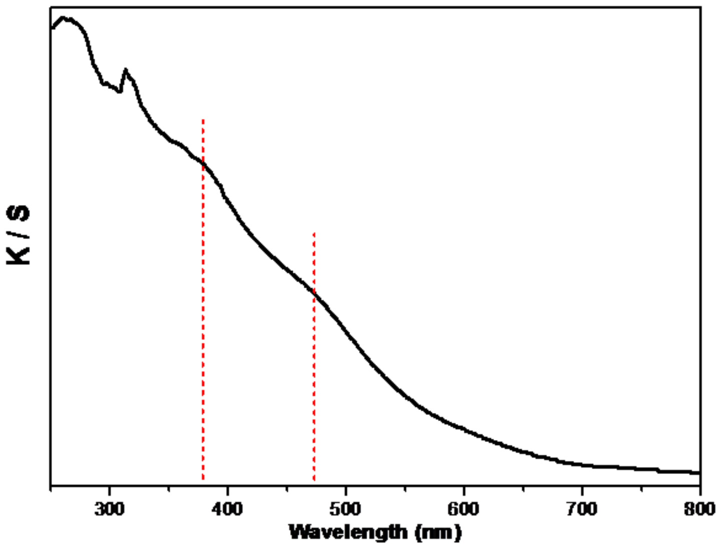

Figure S45. UV/vis diffuse reflectance spectrum of Ir(cod)-$BPy_{0.3}$-NT after the 10th reaction.

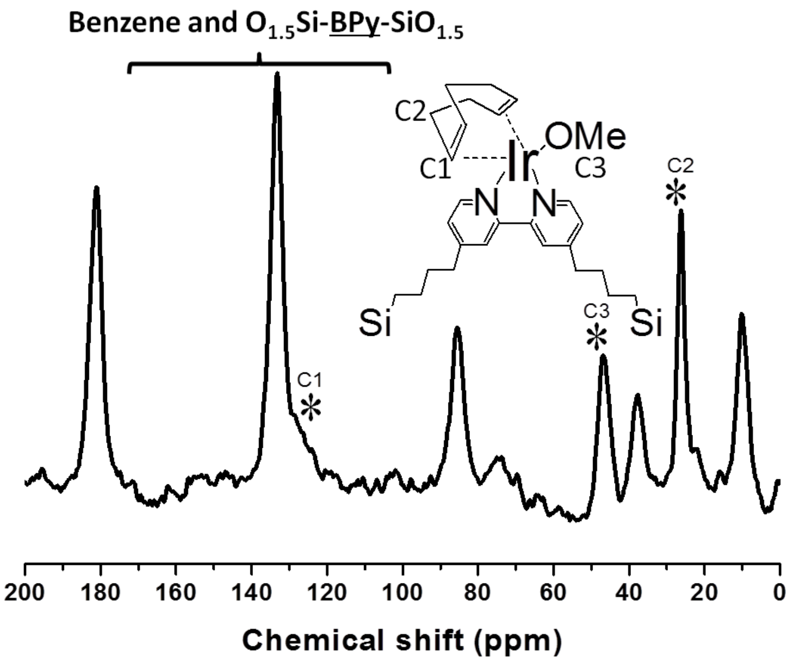

Figure S46. ^{13}C CP MAS NMR spectrum of Ir(cod)-$BPy_{0.3}$-NT after the 10th reaction.

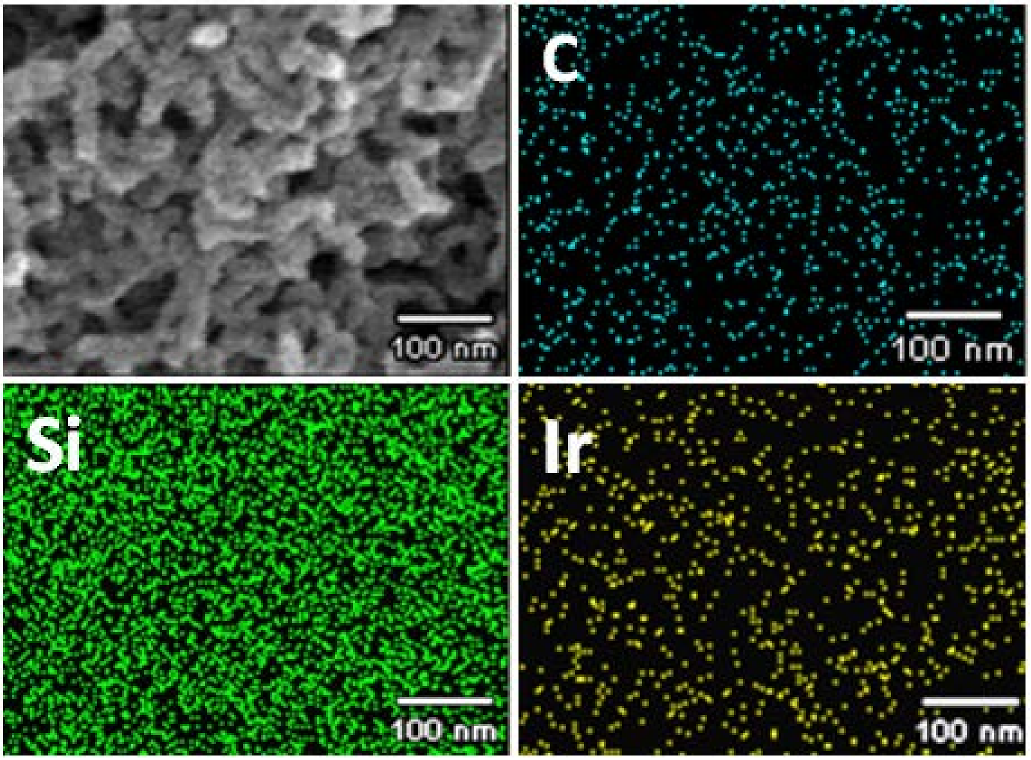

Figure S47. EDX analysis of Ir(cod)-BPy$_{0.3}$-NT after the 10th reaction.

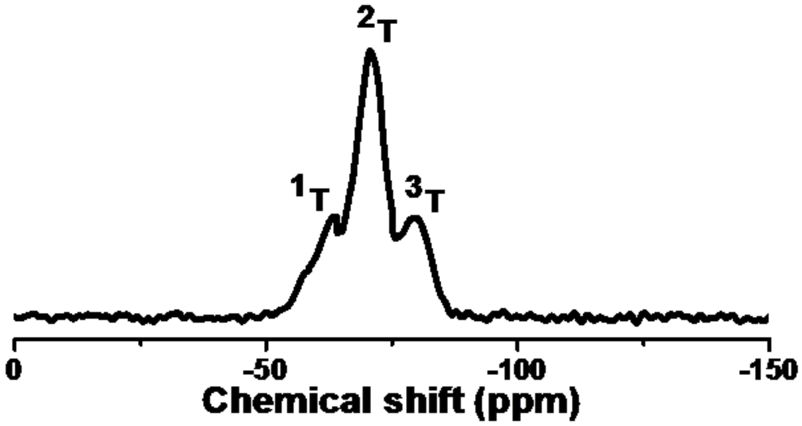

Figure S48. ^{29}Si MAS NMR spectrum of Ir(cod)-BPy$_{0.3}$-NT after the 10th reaction.

14. References

[1] L. Dadci, H. Elias, U. Frey, A. Hoernig, U. Koelle, A. E. Merbach, H. Paulus, J. S. Schneider. *Inorg. Chem.* **1995**, *34*, 306.

[2] M. Waki, Y. Maegawa, K. Hara, Y. Goto, S. Shirai, Y. Yamada, N. Mizoshita, T. Tani, W. J. Chun, S. Muratsugu, M. Tada, A. Fukuoka, S. Inagaki. *J. Am. Chem. Soc.* **2014**, *136*, 4003.

[3] a) X. Liu, X. Li, Z. Guan, J. Liu, J. Zhao, Y. Yang and Q. Yang, *Chem. Commun.* **2011**, *47*, 8073-8075. b) X. Liu, Y. Goto, Y. Maegawa, T. Ohsuna, S. Inagaki, *APL Mater.* **2014**, *2*, 113308.

[4] M. Karaki, A. Karout, J. Toufaily, F. Rataboul, N. Essayem, B. Lebeau, *J. Catal.* **2013**, *305*, 204.
